# Carbon-Flux Distribution within *Streptomyces coelicolor* Metabolism: A Comparison between the Actinorhodin-Producing Strain M145 and Its Non-Producing Derivative M1146

**DOI:** 10.1371/journal.pone.0084151

**Published:** 2013-12-23

**Authors:** Fabien Coze, Françoise Gilard, Guillaume Tcherkez, Marie-Joëlle Virolle, Armel Guyonvarch

**Affiliations:** 1 Unité Mixte de Recherche 8621, Institut de Génétique et Microbiologie, Université Paris-Sud, Orsay, France; 2 Unité Mixte de Recherche 8618, Institut de Biotechnologie des Plantes, Université Paris-Sud, Orsay, France; 3 Centre National de la Recherche Scientifique, Orsay, France; INRA Clermont-Ferrand Research Center, France

## Abstract

Metabolic Flux Analysis is now viewed as essential to elucidate the metabolic pattern of cells and to design appropriate genetic engineering strategies to improve strain performance and production processes. Here, we investigated carbon flux distribution in two *Streptomyces coelicolor* A3 (2) strains: the wild type M145 and its derivative mutant M1146, in which gene clusters encoding the four main antibiotic biosynthetic pathways were deleted. Metabolic Flux Analysis and ^13^C-labeling allowed us to reconstruct a flux map under steady-state conditions for both strains. The mutant strain M1146 showed a higher growth rate, a higher flux through the pentose phosphate pathway and a higher flux through the anaplerotic phospho*enol*pyruvate carboxylase. In that strain, glucose uptake and the flux through the Krebs cycle were lower than in M145. The enhanced flux through the pentose phosphate pathway in M1146 is thought to generate NADPH enough to face higher needs for biomass biosynthesis and other processes. In both strains, the production of NADPH was higher than NADPH needs, suggesting a key role for nicotinamide nucleotide transhydrogenase for redox homeostasis. ATP production is also likely to exceed metabolic ATP needs, indicating that ATP consumption for maintenance is substantial.Our results further suggest a possible competition between actinorhodin and triacylglycerol biosynthetic pathways for their common precursor, acetyl-CoA. These findings may be instrumental in developing new strategies exploiting *S. coelicolor* as a platform for the production of bio-based products of industrial interest.

## Introduction

Since the late 1960s, considerable effort has been devoted to discover new antibiotics. However, the discovery of biologically active molecules has proved progressively more difficult due to biochemically and technically long and costly processes. Furthermore, the prevalence of multi-resistant bacteria has now increased quite substantially [1]. Therefore, discovering novel antibiotics is now seen as critical for public health and medical research programs.

In this regard, the soil-inhabiting, Gram-positive bacteria, belonging to the genus *Streptomyces*, are significant sources of new bio-active molecules [2]. While these bacteria produce up to 70% of antibiotics currently used in medical prescriptions as well as many valuable compounds (such as immune-suppressors or anti-carcinogenic agents), recent publications of the genome sequence from several *Streptomyces* species [3]–[5] highlighted that their potential for the production of new secondary metabolites remains enormous. In fact, genome mining revealed that for a given *Streptomyces* species, the genome comprises 20 to 40 gene clusters presumably involved in the production of many secondary metabolites, while three to five only are currently produced under ordinary laboratory culture conditions.

The genetic organization of genes encoding proteins involved in secondary metabolite production has been studied extensively. Several gene clusters encoding antibiotic biosynthetic pathways, as well as specific or pleiotropic regulators, have now been described [6].

However, although primary metabolism provides precursors, reductive power and energy used for biomass and secondary metabolites biosyntheses, an integrated picture of relationships between primary and secondary metabolism is still missing. A metabolic flux analysis describing carbon flux distribution within the metabolic network, that would complete previous transcriptomic [7] and proteomic studies [8], would clarify the mechanisms by which primary metabolism sustains antibiotic production. This would in turn be of crucial importance for microbiological engineering of *Streptomyces* strains in order to define conditions under which the accumulation of precursors, preferential expression of the above-mentioned gene clusters, or antibiotic synthesis are optimal.

*Streptomyces coelicolor* A3 (2) M145 is a model strain often used to investigate the regulation of antibiotic production and its genome was the first one to be sequenced and annotated amongst *Streptomyces* species [3]. Depending on growth conditions, this strain may produce up to four antibiotics, but generally synthesizes mainly the blue pigmented, polyketide antibiotic actinorhodin as the major product [9].

Considering the production yield of *S. coelicolor* M145, which presumably indicates an efficient synthesis of the polyketide precursor acetyl-CoA, *S. coelicolor* M145 has been chosen as a ‘genetic platform’ to create a super-host for the heterologous expression of gene clusters associated with secondary metabolism from other *Streptomyces* species as well as organisms from other genera [10]–[12]. In order to avoid competition between native and heterologous biosynthetic pathways for a limited pool of metabolic precursors, the four major antibiotic clusters of *S. coelicolor* M145 were deleted, yielding strain M1146 [11].

In *S. coelicolor* M145, production of the Calcium-Dependent Antibiotic (CDA) coincides with growth [13], whereas production of actinorhodin, undecylprodigiosin and methylenomycin usually take place at the onset of the stationary phase when growth rate has slowed down [14], [15]. In the case of actinorhodin, the shift between the growth and the production phases is accompanied by a series of transcriptomic and proteomic switches leading to carbon redirection to actinorhodin synthesis [7], [8], [16].

In the present paper, we developed a N-limited minimal medium to study *S. coelicolor* metabolic fluxes under steady-state conditions. N-limitation was chosen as a typical condition promoting actinorhodin production by *S. coelicolor* M145, as previously described [17]–[19]. Under such conditions, both M145 and M1146 strains grow exponentially at a very low growth rate (*μ*∼0.04 h^−1^), M145 producing only actinorhodin during its growth phase.

We took advantage of metabolic steady conditions to compute a carbon flux distribution in *S. coelicolor* using a combination of stoichiometric inventory described by Holms [20], enzyme activity assays, mass-balance (Metabolic Flux Analysis) and ^13^C isotopic methods (^13^C Metabolic Flux Analysis). Data from genome [3], transcriptome [7] and proteome [8] analyses were used to constraint and define operating metabolic pathways.

We elucidate the flux distribution in primary carbon metabolism in both *S. coelicolor* M145 and M1146 strains and compare them so as to gain insights on effects of the impairment of secondary metabolism on metabolic homeostasis.

## Materials and Methods

### Biological material

The present study was carried out with *S. coelicolor* A3 (2) strain M145 and *S. coelicolor* A3 (2) strain M1146 (*act*, *red*, *cpk*, *cda* deletions). *S. coelicolor* strain A3 (2) M1146 cannot produce actinorhodin (ACT), undecylprodigiosin (RED) and calcium dependent antibiotics (CDA) and no cryptic type I polyketide synthase (Cpk) was expressed. Both strains were a generous gift from M.J. Bibb and J.P. Gomez-Escribano from the John Innes Centre (Norwich, UK).

### Media and growth conditions

Batch cultures were grown in a bioreactor (Infors Labfors 3) with a 2 litre working volume, 2 Rushton-type impellers and 3 baffles. The bioreactor was equipped with a cooled condenser to avoid medium evaporation. Temperature and pH were controlled at 28°C and 7.0, respectively, with automatic addition of NaOH (1 mol L^−1^) or HCl (0.5 mol L^−1^). CO_2_ and O_2_ concentrations were monitored with a Bluesens BCpreFerm gas analyzer. Aeration was adjusted to 0.4 volume air (volume culture)^−1^ min^−1^ (vvm) and agitation was 800 rpm so as to maintain dissolved oxygen near 70% saturation (aerobic conditions).

A defined minimal medium (File S1) was developed according to Egli and Fiechter [21]. Based on theoretical excess factors for mineral elements and vitamins with respect to carbon [21] the medium, although N-limited, allowed *S. coelicolor* strains to reach a bacterial density of 9.6 g (cell dry weight) L^−1^ and to grow for more than 5 generations in the exponential growth phase. Note that exponential growth during at least 5 generations was a prerequisite for steady-state isotope labeling in metabolites [22]. Isotopic labeling was conducted with a mixture consisting of 80% unlabelled glucose and 20% [1-^13^C] glucose (CortecNet). For fermentation experiments, 100 mL of medium no. 1 (15.0 g L^−1^ glucose, 15.0 g L^−1^ glycerol, 15.0 g L^−1^ bacto peptone (Difco 0118-07), 3.0 g L^−1^ NaCl, 1.0 g L^−1^ CaCO_3_, pH 7.2) in a baffled polycarbonate 500-mL Erlenmeyer flask were inoculated with 10^9^ spores from a 30% glycerol stock. The culture was incubated at 28°C and agitated with a magnet (NuovaII agitator, Bioblock Scientific/Sybron thermolyne) for 48 h. Eight mL were then used to inoculate 100 mL of medium no. 2 (33.0 g L^−1^ glucose, 15.0 g L^−1^ yeast extract (BD 212720), pH 7.2). The second culture was incubated for 24 h. Cells from 40 mL were collected by centrifugation (3,200 *g*, 5 min, room temperature), suspended in 20 mL of TES solution (270.5 mmol L^−1^), and immediately used to inoculate the bioreactor. An additional sample of 10 mL was used for cell dry weight quantification, after filtration through a cellulose acetate filter (0.22 µm) and distilled water washing before drying at 95°C for 24 h.

### Gas exchange

In bioreactors, liquid and gas phases were well mixed and gas diffusion was rapid such that both the CO_2_ Exhaust Rate (*CER*) and the O_2_ Uptake Rate (*OUR*) could be calculated at each time with online measurements: 
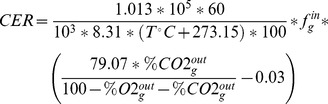


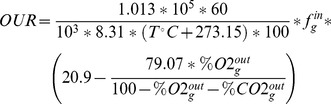



In these equations, it is assumed that the build-up of dissolved CO_2_ and N_2_ in the liquid phase was negligible. Flows (*f_g_^in^*) as well as mole fractions (% CO_2_, % O_2_) were measured with dehydrated air to avoid any mole fraction imbalance due to water vapour. *CER* and *OUR* were plotted versus time and summed in order to quantify total CO_2_ evolution and O_2_ consumption. The latter values are used in section Materials and methods 12.1. to determine *Y_CO2/S_* and *Y_O2/S_* respectively. All the abbreviations and symbols used in this study are listed in File S2.

### Nucleic acids, proteins, antibiotics, triacylglycerol measurements and elemental composition

Nucleic acids were quantified after Norris and Ribbons [23] and proteins were quantified with the Bradford method [24]. Actinorhodin and undecylprodigiosin contents were determined spectrophotometrically according to Christiansen [25] and Tsao *et al*. [26] respectively. Production of CDA was detected using a bioassay adapted from Lautru *et al.* [27]. Triacylglycerol contents were determined by Fournier Transformed Infra Red Spectroscopy (FTIR) as described by Verleyen *et al.* [28] and Hong *et al*. [29]. FITR analyses were carried out at the Laboratoire de Chimie Chimie Physique, UMR 8000, CNRS/Université Paris Sud, Orsay, France (see File S1 for further details). The elemental composition (C, H, O, N) of *S. coelicolor* was determined with an elemental analyser (VarioCube, Elementar) on cells harvested during the exponential phase of growth. Elemental analyses were carried out at the Institut de Chimie des Substances Naturelles, UPR 2301, CNRS, Gif sur Yvette, France.

### Proteins precipitation for metabolite analyses

Supernatants were de-proteinated by acid precipitation with HClO_4_ 35% (0.1 mL (mL supernatant) ^−1^) and then centrifuged 2 min at 6,000 *g* at room temperature. Supernatants were neutralized with cold KOH (7 mol L^−1^), centrifuged 2 min at 6,000 *g* at room temperature and filtered through a 0.22 µm polyether sulfone membrane.

### Glucose and extracellular metabolites quantification

Glucose, α-ketoglutarate (2-oxoglutarate), pyruvate, L-lactate, acetate and ethanol were analysed from de-proteinated supernatants on an Agilent 1200 HPLC instrument with an Aminex HPX-87H column at 35°C using H_2_SO_4_ (5 mmol L^−1^) as the eluent (0.5 mL min^−1^). Metabolites were detected either with an Agilent SPD UV detector (210 nm) or an Agilent RID detector (40°C).

### Ammonium quantification

The ammonium content was measured enzymatically in 20 µL of de-proteinated samples with glutamate dehydrogenase in 1 mL of an assay mixture made of Tris/HCl (0.1 mol L^−1^) pH 8.0, NADH (0.2 mmol L^−1^), ADP (0.6 mmol L^−1^), α-ketoglutarate (10 mmol L^−1^), and 5 units of glutamate dehydrogenase from bovine liver (Sigma #G2626). The *A*_340_ was monitored until the end of the reaction (∼8 min).

### Phosphate quantification

Phosphate was measured on 50 µL from de-proteinated samples using 100 µL of PiBlue (PiBlue Phosphate Assay Kit, Gentaur). The *A*_600_ was monitored for 30 min at room temperature.

### Enzymatic assays

40 mL of culture were harvested and bacterial cells were suspended in 10 mL Tris-Tricarballylic acid (15 mmol L^−1^) buffer pH 7.8, MgCl_2_ (10 mmol L^−1^), 10% glycerol. RNase A (1 mg mL^−1^) and DNase I (1 mg mL^−1^) were added when needed. The sample was sonicated 8 times with a Branson Sonifier 250 sonicator set at a power level  =  2, during 20 seconds at 30 seconds intervals. Cell debris was removed by centrifuging at 10,000 *g* for 15 min at 4°C. The supernatant was used as the crude extract. The protein concentrations were measured by the Bradford method using BSA as a standard [24]. All enzyme activities were measured on a Beckmann DU7400 spectrophotometer at 28°C according to Sugimoto and Shiio [30], [31], Nachlas *et al*. [32] and Sauer *et al*. [33] with slight modifications (see File S1 for further details).

### Sample preparation and GC-MS analyses

GC-MS analyses of amino acids to determine the ^13^C-labeling (see File S1 for further details) were carried out as described by Zamboni *et al*. [34]. Briefly, after 5 generations of exponential growth in the presence of 20% [1-^13^C] glucose, cells were collected, proteins were extracted and submitted to acidic hydrolysis, extracts were derivatized with methoxamine and N-methyl-N-(trimethylsilyl) trifluoroacetamide and injected on a GC-MS (Agilent 6890N gas chromatograph, time-of-flight mass spectrometer Pegasus III). Peak integration was performed using LECO Pegasus software. Because automated peak integrations have been occasionally unreliable, manual controls and/or corrections were systematically performed for each metabolite.

Five independent samples were collected along a 1.5-generation period and analysed in triplicate. During the sampling period, *μ*, *q_S_*, *q_O2_*, *γ_CO2_*, *Y_X/S_*, *Y_ACT/S_*, *Y_CO2/S_* and *Y_O2/S_* values were constant and at their maximal values. MID values for Glu, Thr, Asp, Ala and Val were identical, with less than 1% RDS. We therefore conclude that both metabolic and the isotopic steady-states were ensured in our experiment.

### *S. coelicolor* central metabolism

#### Pathways considered

The central metabolic network of *S. coelicolor* was deduced primarily from genome annotation [3], transcriptome [7] and proteome analyses [8].

Glucose entry and phosphorylation through the Glk-GlcP permease complex in *S. coelicolor* were well described [35]–[37]. Consequently, we assumed that glucose phosphorylation was carried out by the ATP-dependent glucose kinase Glk.

All genes and enzymes involved in the Embden-Meyerhof-Parnas pathway (EMP), the tricarboxylic acid cycle (TCA), and the pentose phosphate pathway (PPP) were detected in *S. coelicolor* [8], [38]–[40].

The Entner-Doudoroff pathway (ED) has been shown to be non-functional in several *Streptomyces* species [39], [41]. Since *(i)* there was little growth with two carbon substrates and *(ii)* both isocitrate lyase and malate synthase were not detected in the *S. coelicolor* proteome [8], the glyoxylate shunt was considered to be negligible in our model.

In the present paper, comparison of the experimental and calculated *γ_CO2_* values, and analysis of ^13^C-labeling in valine, alanine, glutamate, glutamine and proline provided further evidence of these assumptions for *S. coelicolor* under our experimental conditions (see 3.4.).

PEP carboxylase was shown to be the sole anaplerotic enzyme in *Streptomyces* species [8], [38], [39], [42], [43] and was therefore considered in our model.

Few experimental data on the respiratory chain efficiency in *Streptomyces* species are available [44]. From consideration of genome mining, we assumed here that the respiratory chain of *S. coelicolor* could be branched, with a type I NADH dehydrogenase excreting 4 protons per NADH, an uncoupled type II NADH dehydrogenase and two terminal oxidases, the *bd* complex that excretes 2 protons per NADH and the *bc_1_/aa_3_* supercomplex that excretes 6 protons per NADH [45]. The type I NADH dehydrogenase has been shown to account for about 70% of NADH dehydrogenase activity in aerobic conditions in *Escherichia coli* [46]. The supercomplex *bc_1_/aa_3_* was shown to have a lower affinity for oxygen and to be the predominant complex in aerobic conditions [47]. Enzymes corresponding to both type I NADH dehydrogenase and to *bc_1_/aa_3_* supercomplex were identified in *S. coelicolor* proteome [8]. Genome mining and proteome analyses [8] further revealed the presence of an F_1_F_0_-ATP synthase. Under the assumption that the synthesis of one ATP by F_1_F_0_-ATP synthase requires three to four protons, NADH re-oxidation via the *bc1/aa3* branch yields 1.50–3.33 ATP. FADH re-oxidation was assumed to be due to succinate dehydrogenase [8], [44]. Depending on the pathway used by electrons, the transfer of two electrons from succinate to oxygen yields 0.50–2.00 ATP [48].

Considering all these data, a P/O value (moles of ATP produced per 0.5 mole of O_2_ consumed) of 2.0 could be reasonably considered for *S. coelicolor*. Nevertheless, our results on carbon flux distribution are not sensitive to the P/O value used.

Two genes of *S. coelicolor* (*SCO7622* and *SCO7623*) putatively encode a PntAB-type nicotinamide nucleotide transhydrogenase that interconverts NADP + NADH to NADPH + NAD in *E. coli* [33], and thus the presence of a transhydrogenase was taken into account.

#### Amino acids, nucleic acids, peptidoglycan, lipids and small molecules biosyntheses

Amino acids, nucleic acids, small molecules, peptidoglycan, triacylglycerol and lipids biosynthetic pathways in *S. coelicolor* appeared to be identical to those present in other bacteria [42] and therefore their stoichiometry was assumed to be the same as that observed in other microorganisms [22], [49]–[61] (see File S3).

#### Antibiotic biosyntheses

The biosynthetic pathways for the 3 known antibiotics produced by *S. coelicolor* were reconstructed using *S. coelicolor* genome and published data [62]–[70]. For each antibiotic, all the steps of the biosynthesis were fused into one single reaction that includes precursors, reducing power and energy needs.

#### Biomass composition

To calculate carbon fluxes according to the stoichiometric approach of Holms [20], the biomass composition of *S. coelicolor* cells had to be determined. The respective amounts of the main polymers involved in the *S. coelicolor* biomass composition, namely proteins, DNA, RNAs, triacylglycerol, lipids and peptidoglycan were taken from Borodina *et al*. [71] with some modifications according to the experimental values we determined. These modifications are discussed in section Results 1.

#### Precursors, cofactors and energy requirements

Using the biomass composition, reactions associated with building-block biosynthesis (primary metabolism) and reactions for secondary metabolism, we calculated precursor, cofactor and energy requirements to synthesise 1 g of dry cells. On the basis of Ingraham's calculations for *E. coli* [22], the energy requirement for the polymerisation of macromolecules in *S. coelicolor* was calculated. We also calculated specific precursor, energy and reducing power requirements to synthesize 1 mole of antibiotics.

### Flux calculations

#### Experimental kinetic parameter quantifications

For each experiment, we calculated the following graphs: *ln(X(t))* versus *t*, *X(t)* versus *S(t)*, *ACT(t)* versus *S(t)*, *RED(t)* versus *S(t)*, *KET(t)* versus *S(t)*, *PYR(t)* versus *S(t)*, *CO_2_(t)* versus *S(t)*, *O_2_(t)* versus *S(t)*, where *X* denotes biomass and *S* denotes source glucose. During exponential growth, each curve was linear, and the slope allowed us to determine growth rate constant *μ* (h^−1^) and the following yields: *Y_X/S_*, *Y_ACT/S_*, *Y _RED/S_*, *Y_CDA/S_*, *Y_KET/S_*, *Y_PYR/S_*, *Y_CO2/S_*, *Y_O2/S_*. For example, *Y_X/S_* is the yield of biomass production with respect to glucose, etc. Note that yields are expressed in carbon equivalents, *e.g. Y_X/S_* is in moles of biomass carbon per mole of glucose carbon. Using these values, the glucose consumption and productivity of metabolites (μmol (g cell dry weight)^−1^ h^−1^) were computed as (note that the following are algebraic and therefore can be negative):
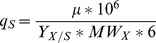


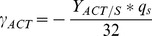


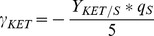


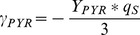


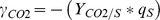


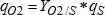



Carbon balance (in %) was calculated as follows:
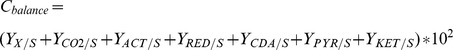



#### Anabolic fluxes calculation

Glucose-6-phosphate, fructose-6-phosphate, ribose-5-phosphate, erythrose-4-phosphate, glyceraldehyde-3-phosphate, 3-phosphoglycerate, phospho*enol*pyruvate, pyruvate, acetyl-CoA, α-ketoglutarate and oxaloacetate are the 11 precursors from central metabolism. These precursors are sufficient to synthesize any macromolecule in the bacteria. That is, carbon flux to anabolic reactions can be represented by 11 individual fluxes from precursors to biomass and/or secreted metabolites (antibiotics, organic acids). Known molar requirements of biomass or antibiotic for precursors (for a given precursor *i*, they are denoted as *p_i_* for biomass, *l_i_* for actinorhodin, *m_i_* for undecylprodigiosin, *n_i_* for the calcium dependent antibiotic expressed in μmol (g cell dry weight)^−1^ and μmol (μmol antibiotic)^−1^, respectively) and productivity (*q* and *γ* values, see Materials and Methods 12.1) allow one to calculate anabolic requirement fluxes *Φ_i_* and the sum *v_i_* for any precursor *i*, as follows: 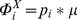
(biomass)

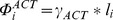
(actinorhodin)

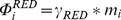
(undecylprodigiosin)

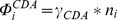
(calcium)

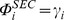
(organic)

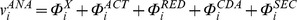



#### Metabolic Flux Analysis

Although anabolic fluxes *Φ_i_* were calculated as described in Materials and Methods 12.2, catabolic fluxes in central metabolism calculations remained to be determined to obtain a full carbon-flux distribution. For this purpose, metabolic reactions were represented as a stoichiometric matrix (***S***) of size {m, n}. Each row of this matrix represented one unique compound amongst the m = 48 compounds and each column represented one reaction amongst the n = 46 lumped metabolic reactions. Note that the 46 lumped reactions correspond to 265 enzymatic reactions. ***S*** was a sparse matrix, with 0 as the most represented value. The flux through all reactions in the metabolic network was represented by the vector ***v*** (dimension n). The set of concentrations in metabolites was represented by the vector ***x*** (length m). The system dynamics is described by *d* ***x*** */dt* = ***Sv***. In the steady state, *d* ***x*** */dt* = 0, that is, ***Sv*** = 0. In the present study, this equation is true as metabolism was studied at the steady state, that is, in the exponential growth phase in which turn-over fluxes are constant and therefore there is no build-up in intracellular metabolite concentration. Glucose specific consumption (*q_S_*) and the 11 anabolic fluxes (*Φ_i_*) are used as external constraints. However, the system still has one degree of freedom. Data from labeling experiments were used to solve the system. O_2_ consumption, CO_2_ evolution and the respiratory quotient (*QR*) were not used as constraint here and were rather compared to the computed O_2_ and CO_2_ fluxes to check the reliability of our system, as discussed in section Results 2.

#### ^13^C Metabolic Flux Analysis

Here, we took advantage of the software OpenFlux, which is a computational tool based on the Elementary Metabolite Units (EMU) framework [72], [73] to analyse ^13^C-labeling data. The EMU framework used here for the simulation is provided in Table S1. OpenFlux calculates fluxes from Mass Isotopic Distribution (MID, or isotopic pattern) of amino acids. [^13^C-1] glucose was used here as the labeling substrate and allowed us to determine the flux-ratio between the pentose phosphate pathway and glycolysis (PPP/Glyc ratio). In fact, when [^13^C-1] glucose was metabolized by the PPP, the ^13^C label was lost by decarboxylation, while the ^13^C label was incorporated into amino acids when it enters glycolysis. We recognize that reversible triose phosphate isomerase and aldolase activities could lead to a ^13^C-labeling in the C-6 atom position in fructose-1, 6-bisphosphate and then possibly in hexose phosphates [74]. If significant, this effect should be taken into account to calculate the relative commitment of PPP and glycolysis. The ^13^C-labeling in tryptophan and histidine should provide evidence of the labeling in C-6 because their -COOH group comes from this very atom position. While tryptophan was absent from the spectra since it is degraded to a considerable extent by acid hydrolysis [75], the ^13^C-labeling pattern in histidine (Table S2) showed that the C-6/C-1 isotopic exchange is negligible. Thus, the output of our calculations represent actual net fluxes.

MID data from mass-spectrometer were normalized to total mass-signals as follows, giving relative isotopic abundance:
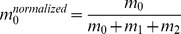


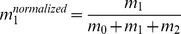


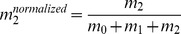



Normalized MID were also corrected for natural isotope (C, H, N, O, Si) abundance during flux calculations. The glucose specific consumption *q_S_*, the 11 anabolic fluxes *Φ_i_* and the normalized MID in glutamate (*m*_0_/*z* = 348), threonine (*m*_0_/*z* = 320), aspartic acid (*m*_0_/*z* = 334), alanine (*m*_0_/*z* = 218) and valine (*m*_0_/*z* = 246) were combined to provide a unique solution for all carbon fluxes, thereby solving vector ***v***.

For sensitivity analyses, carbon fluxes were estimated by minimizing the variance-weighted sum of squared residuals (SSR) between the simulated and model-predicted MIDs using non-linear least-squares régression [76]. In all cases, flux estimation was repeated at least 20 times starting with random initial values for all fluxes to find a global solution. At convergence, standard deviations and 95% confidence intervals for all fluxes were calculated using the parameter continuation technique described by Antoniewicz *et al*. [76]. This technique is based on evaluating the profile of SSR as a function of one flux, while the values for the remaining fluxes are optimized. The 95% confidence interval for an evaluated flux corresponds to flux values for which SSR increased by less than 3.84 [76]. A flux was considered non-observable if the 95% confidence interval was larger than the estimated flux value.

Metabolic fluxes were expressed as absolute (μmol (g cell dry weight)^−1^ h^−1^) or relative fluxes with respect to glucose uptake (μmol (μmol glucose) ^−1^). Relative fluxes allowed the comparison between strains, as M145 and M1146 did have different glucose uptake rates.

#### Energy, reducing power and gas exchange fluxes calculations from carbon-flux distribution

With the formula used for precursors, the stoichiometry of reactions, including those for antibiotics syntheses (File S3), and the flux values (Materials and Methods 12.4), it was possible to calculate ‘intrinsic energy production’ (ATP), reducing power (NADH, NADPH) and gas exchange (O_2_, CO_2_). The total CO_2_ evolution rate was computed from catabolic CO_2_ production (glycolysis, TCA, PPP) and anabolic production as: 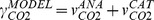



Then, this value was compared with our experimental CO_2_ production rate to verify the validity of the carbon-flux distribution (see section Results 4). In other words, experimental CO_2_ evolution was an independent data point not used as a constraint to calculate carbon-flux distribution. We assumed that NADPH requirement and production should have been equal since the metabolism was in steady state. If the production of NADPH was more important than the need, excess NADPH might have been converted to NADP^+^, thereby reducing NAD^+^ into NADH, by nicotinamide nucleotide transhydrogenase.

Similarly, the metabolic requirement for NADH should match NADH production; but here and quite generally, NADH production was larger than NADH oxidation. In order to satisfy our steady state condition, we assumed that excess NADH was re-oxidized by the respiratory chain. For O_2_ consumption, the anabolic demand was completed by the extra consumption required for excess NADH re-oxidation with the following stoichiometry: 1 mole of re-oxidized NADH consumed 0.5 mole of O_2_. For ATP as well, anabolic and catabolic fluxes were completed by the production of ATP associated with excess NADH re-oxidation. At this stage, knowledge of the actual P/O value (moles of ATP generated per mole of oxidized NADH) would be ideal. As stated in section Materials and Methods 11.1, we assumed that under 70% dissolved O_2_, the *bc_1_/aa_3_* cytochrome pathway probably prevailed, and so a P/O value of 2 could be used to estimate ATP production from oxidative phosphorylation. Under this assumption, as for NADH, ATP production would be higher than ATP consumption. Excess ATP production would be likely compensated for by the consumption required for cell maintenance. Such a consumption is hereafter denoted as mATP, that is, energy requirements for homeostasis and turn-over of structural molecules as well as others processes that could not be quantified precisely (*e.g.* energy needed to excrete antibiotic).

## Results

### Composition of *S. coelicolor* biomass

As mentioned in section Materials and Methods 11.4, we mainly used here metabolite contents reported by Borodina *et al*. [71] with significant modifications for deoxynucleotides (for reasons that will become apparent below), the inclusion of bactoprenol (0.34 µmol (g cell dry weight)^−1^) in cell wall composition [50], and a modification of the triacylglycerol content in M1146. The biomass decomposition in building blocks is shown in Table 1. Using the elemental formula of individual metabolites and taking into account the loss of water during polymerization of macromolecules, the building block composition allowed us to recalculate the macromolecular composition of bacteria in proteins, DNA, RNAs and lipids (Table 2). Our estimates were in agreement with published values [77]–[80] except for phospholipids and DNA.

**Table 1:** Biomass composition of *S. coelicolor* M145 cells (μmol (g dry cells)^−1^).

PROTEINS		PHOSPHOLIPIDS		SOLUBLE MOLECULES	
Alanine	556	Phosphatidylethanolamine	29	NAD	6
Arginine	145	Phosphoglycerol	6	NADP	5
Asparagine	162	Cardiolipin	1	CoA	5
Aspartate	160	C14	10	Menaquinone 8	4
Cysteine	68	C15	31	Tetrahydrofolate	8
Glutamate	157	C16	28	FMN	8
Glutamine	156	C17	31	FAD	5
Glycine	830	C18:1	6	ATP	1.5
Histidine	54	TRIACYLGLYCEROLS		ADP	0.5
Isoleucine	196	Glycerol-3-Phosphate	22	PEPTIDOGLYCAN	
Leucine	305	C14	1	UDP-NAM^b^	110
Lysine	205	C15	30	UDP-NAG^a^	132
Methionine	91	C16	7	Alanine	209
Phenylalanine	98	C17	28	Diaminopimelic acid	125
Proline	173	C18:1	0.2	Glutamate	11
Serine	189	TEICHOIC ACIDS		Glycine	107
Threonine	191	Teichoic acid	16	Bactoprenol Phosphate	0.3
Tryptophan	24	Lysine	4	DNA	
Tyrosine	65	RNA		dAMP	27
Valine	328	AMP	100	dCMP	70
CELL WALL		CMP	172	dTMP	27
UDP-NAG^a^	83	TMP	110	dGMP	70
Galactose	167	GMP	138		

a UDP-N-Acetylglucosamine

b UDP-N-Acetylmuramic acid

**Table 2:** Macromolecules content of *S. coelicolor* M145 cells.

Macromolecules	Published data^a^	Calculated data^b^	RSD^c^
Proteins	41.2	41.1	0.17
DNA	3.6	6.0	35.36
RNA	16.7	16.7	0.00
Phospholipids	2.7	3.3	14.14
Triacylglycerols	1.8	2.0	7.44
Soluble molecules	3.0	2.6	10.10
Peptidoglycan	11.0	11.4	2.53
Cell Wall	4.4	4.4	0.00
Teichoic acids	6.6	6.8	2.11
Ash	9.0	5.7	31.75
Total	100.0	100.0	0.00

a Biomass composition (% dry cell weight) from [71].

b Calculated from our composition of building blocks (% dry cell weight)

c Relative standard deviation (%) between published and calculated values.

The difference in phospholipid content was considered to be acceptable (0.42% of total biomass) since it was previously shown that such a low difference in biomass composition had a negligible impact on carbon flux determination [81].

The experimental DNA content obtained here was 6.0% (0.06 g (g cell dry weight)^−1^). As the mass of the *S. coelicolor* genome is 1.86-fold that of *E. coli*, which has a well-accepted DNA content of 3.1% [82], the value of 6% is much more accurate than that previously published for *S. coelicolor* [79].

The chemical elemental formula calculated from the data in Table 1 (with a correction for water content from 4 to 5% H_2_O excess [83]) was CH_1.62_ O_0.45_ N_0.25_ (normalized with respect to carbon), with 5.7% of ash. This is close to those found in other microorganisms [78], [84]–[87] and in good agreement with the elemental formula determined experimentally by combustion/reduction-based elemental analysis. We thus considered that the biomass composition described in Table 1 was accurate.

A difference in the triacylglycerol content was found in the biomass composition of M145 and M1146, with a triacylglycerol content of 0.02 g (g cell dry weight)^−1^ for M145 and 0.03 g (g cell dry weight)^−1^ for M1146 (M. Vergne, personal communication).

Using (*i*) the biomass composition (Table 1, Table 2), (*ii*) the stoichiometry of reactions describing the biosynthesis of metabolites (File S3) and (*iii*) the energy requirements re-calculated for *S. coelicolor* on the basis of published energy requirements for the polymerisation of macromolecules in *E. coli* [22], we calculated precursors, cofactors and energy requirements to synthesise 1 g of dry cell matter (Table 3).

**Table 3:** Precursors, energy and reducing power needs for 1 g dry biomass synthesis in *S. coelicolor*.

Precursors, Energy, Reducing Power	μmol (g cell dry mass)^−1^	
	M145	M1146
Glucose-6-phosphate	327	327
Fructose-6-phosphate	357	357
Ribose-5-phosphate	1,344	1,344
Erythrose-4-phosphate	199	199
Glyceraldehyde-3-phosphate	281	292
3-Phosphoglycerate	1,701	1,701
Phosph*enol*pyruvate	508	508
Pyruvate	2,600	2,629
Acetyl Coenzyme A	2,043	2,234
α-Ketoglutarate	754	754
Oxaloacetate	2,773	2,802
CO_2_	−1,433	−1,491
O_2_	17	17
ATP	34,606	34,849
NADPH	13,491	13,993
NADH	−3,387	−3,415

Positive values correspond to metabolite consumptions and negative values correspond to metabolite productions.

### Enzymatic activities in *S. coelicolor*

In *Streptomyces lividans* and other Actinomycetes, glucose-6-phosphate dehydrogenase was shown to be NADP-dependent whereas 6-phosphogluconate dehydrogenase was shown to be NAD-dependent [39], [88]. Because of its importance for redox mass-balance, the nature of the cofactors was verified here. We found that glucose-6-phosphate dehydrogenase was strictly NADP-dependent, with a specific activity of 144.6±2.7 µmol min^−1^ (mg protein)^−1^ and no detectable activity with NAD. Furthermore, we found that 6-phosphogluconate dehydrogenase was strictly NADP-dependent, with a specific activity of 236.4±6.4 µmol min^−1^ (mg protein)^−1^ and no detectable activity with NAD. We also verified that isocitrate dehydrogenase was strictly NADP-dependent, with a specific activity of 132.7±4.3 µmol min^−1^ (mg protein)^−1^ and no activity at all with NAD.

The *S. coelicolor* genome contains two genes (*SCO7622* and *SCO7623*) encoding a PntAB-like nicotinamide nucleotide transhydrogenase, but no UdhA-encoding gene. In *E. coli*, PntAB reduces NADH using NADP as a reductant *in vivo* while UdhA catalyses the reverse reaction [33]. By enzymatic measurement, we found a clear transhydrogenase activity, that catalysed the reduction of thio-NAD using NADPH with a specific activity of 90.4±3.7 µmol min^−1^ (mg protein)^−1^, and no activity at all with thio-NADP and NADH. Since no evident NADPH phosphatase-encoding gene was found in the *S. coelicolor* genome, transhydrogenase was then introduced in our model as the enzyme that balanced NADPH overproduction.

### Growth and metabolite excretion of *S. coelicolor* strins

Batch-cultivation of the strains M145 and M1146 yielded a bacterial density of 8 g (cell dry weight) L^−1^ and 9 g (cell dry weight) L^−1^, respectively. The stationary growth phase began when the medium turned to be nitrogen-depleted, glucose and phosphate being limiting approximately 9 h after nitrogen depletion for both strains.

During the exponential phase in bioreactors, the growth rate constant (*μ*) of M1146 was 13±0.1% higher compared to M145 while the glucose specific uptake rate was nearly 13±0.5% lower (Table 4). The respiratory activity (O_2_ consumption and CO_2_ production) was 20±2% lower in M1146 compared to M145 and furthermore, strain M145 produced 2.03±0.08 µmol (g cell dry weight)^−1^ h^−1^ actinorhodin (up to 400 mg L^−1^ at the end of the culture) while no antibiotic production was detected at all in M1146.

**Table 4:** Kinetic parameters and GC-MS analyses.

	*S. coelicolor* M145		*S. coelicolor* M1146	
	Values	RSD (%)	Values	RSD (%)
*μ* (h^−1^)	0.0394	0.54	0.0446	0.95
				
*q*_S_ μmol (g cell dry mass)^−1^ h^−1^	664	3.24	575	1.40
*q_O2_* μmol (g cell dry mass)^−1^ h^−1^	2,235	2.97	1,528	6.67
*γ_CO2_* μmol (g cell dry mass)^−1^ h^−1^	−2,295	3.24	−1,372	0.86
*γ_ACT_* μmol (g cell dry mass)^−1^ h^−1^	−2	5.87	0	0.00
				
Glu-TMS (*m/z* = 348) *m*_0_	0.54614	0.24	0.57610	0.30
Glu-TMS (*m/z* = 349) *m*_1_	0.30993	0.79	0.28750	0.34
Glu-TMS (*m/z* = 350) *m*_2_	0.14393	0.97	0.13640	0.83
Thr-TMS (*m/z* = 320) *m*_0_	0.57694	0.11	0.60627	0.14
Thr-TMS (*m/z* = 321) *m*_1_	0.29529	0.31	0.27454	0.45
Thr-TMS (*m/z* = 322) *m*_2_	0.12776	0.53	0.11919	0.98
Asp-TMS (*m/z* = 334) *m*_0_	0.57730	0.22	0.60477	0.08
Asp-TMS (*m/z* = 335) *m*_1_	0.29536	0.22	0.27410	0.37
Asp-TMS (*m/z* = 336) *m*_2_	0.12733	0.52	0.12113	0.96
Ala-TMS (*m/z* = 218) *m*_0_	0.71483	0.19	0.72507	0.15
Ala-TMS (*m/z* = 219) *m*_1_	0.20701	0.51	0.19744	0.52
Ala-TMS (*m/z* = 220) *m*_2_	0.07817	1.72	0.07749	0.85
Val-TMS (*m/z* = 246) *m*_0_	0.64474	0.36	0.66780	0.25
Val-TMS (*m/z* = 247) *m*_1_	0.26438	0.74	0.24531	0.70
Val-TMS (*m/z* = 248) *m*_2_	0.09088	0.45	0.08689	0.62

Kinetic parameters were experimental values obtained as described in 2.19.1. Mass Isotopic Distributions were normalized as described in section 2.19.4. Values were means of two independent experiments and RSD their relative standard deviations. Glu: Glutamic acid; Thr: Threonine; Asp: Aspartic acid; Ala: Alanine; Val: Valine.

For *q_S_*, *γ_ACT_*, *q_O2_* and *γ_CO2_*, positive values correspond to metabolite consumption and negative values correspond to metabolite production.

The carbon balance during the exponential phase was similar in both strains (92±2% in M145 and 87±1% in M1146) and slightly lower than 100%, suggesting that a certain carbon amount was not recovered. HPLC, LC-MS analyses and bioassays on culture supernatants did not allow the identification of extracellular metabolites that could account for this apparent loss of carbon. Nevertheless, these carbon balances are similar to the 94±9% value found by Borodina *et al*. [41] for *S. coelicolor* M145 and to the 90% value found by Bruheim *et al*. [89] for *S. lividans* during actinorhodin production in N-limited conditions. The carbon imbalance probably indicates the synthesis of one or several yet unidentified secondary metabolites in *S. coelicolor* under N-limitation.

During the stationary phase, both strains produced α-ketoglutarate (82 µmol±4 (g cell dry weight)^−1^ h^−1^ in M145 and 162 µmol±6 (g cell dry weight)^−1^ h^−1^ in M1146) and pyruvate (21 µmol±1 (g cell dry weight)^−1^ h^−1^ in M145 and 167 µmol±11 (g cell dry weight)^−1^ h^−1^ in M1146). There was, therefore, a clear difference in carbon allocation between the two strains, with M1146 liberating more pyruvate and α-ketoglutarate in the medium, likely as a result of carbon re-direction in the absence of growth and antibiotic production, as discussed in section 4.4.

### Flux patterns of *S. coelicolor* strains

The use of stoichiometric coefficients and biomass composition to infer metabolic fluxes (Metabolic Flux Analysis) could not be carried out properly since an uncertain flux value remained at the PPP/glycolysis branching-point. We thus used ^13^C-labeling ([^13^C-1] glucose) and ^13^C Metabolic Flux Analysis in the isotopic steady-state to estimate the relative commitment to PPP as opposed to glycolysis. Isotopic patterns obtained by GC-MS in amino acids from protein hydrolysates are shown in Table 4. In the amino acids considered (Glu, Thr, Asp, Ala and Val), there was a lower ^13^C-labeling (mass *m*_0_ proportionally more abundant) in the strain M1146, clearly showing a lower commitment of the ^13^C-label to glycolysis and the TCA (from which those amino acids are synthesized) and thus a larger commitment to PPP.

Analysis of ^13^C-label patterns in alanine and valine clearly showed the absence of ^13^C label in the C-1 atom position in both amino acids (Table S2). This demonstrated that the C-1 atom position in pyruvate was not labelled and thus the absence of an active Entner-Doudoroff pathway under our conditions. Moreover, a discrepancy between *γ_CO2_* and 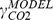 would have been detected if either the Entner-Doudoroff pathway or the glyoxylic shunt had been active in our conditions, but this is clearly not the case (see also below). Similarly, the ^13^C-labeling in C-1 in glutamate, glutamine and proline showed the activity of the anaplerotic phospho*enol*pyruvate carboxylase and examination of the ^13^C-labeling at the C-5 position in glutamate, glutamine and proline indicated that the glyoxylic shunt was inactive (Table S2).

Using further the data of Table 4, fluxes were numerically estimated with OpenFlux, which is based on optimization of weighted least squares (see section Materials and Methods12.4).

We analyzed the statistical significance and confidence of estimated fluxes in *S. coelicolor* M145 and M1146, with [^13^C-1] glucose as a tracer and the sensitivity test described by Antoniewicz *et al.* [76]. Small sensitivities, *i.e*., large changes in the flux value resulting in small changes in the minimized sum of squared residuals, indicate that the flux cannot be estimated precisely. Large sensitivities, on the other hand, indicate that the flux is well determined. In the present study, sensivity tests fulfilled large sensitivity (96%) with a 95% confidence level in all cases. According to Wiechert *et al.* (1997), our flux maps are representative descriptions of the metabolic processes in *S. coelicolor* [90].

In M145 and M1146, the flux through the PPP was found to be 22±0.3% and 38±0.2% of available glucose-6-phosphate, respectively.

**Figure 1:**
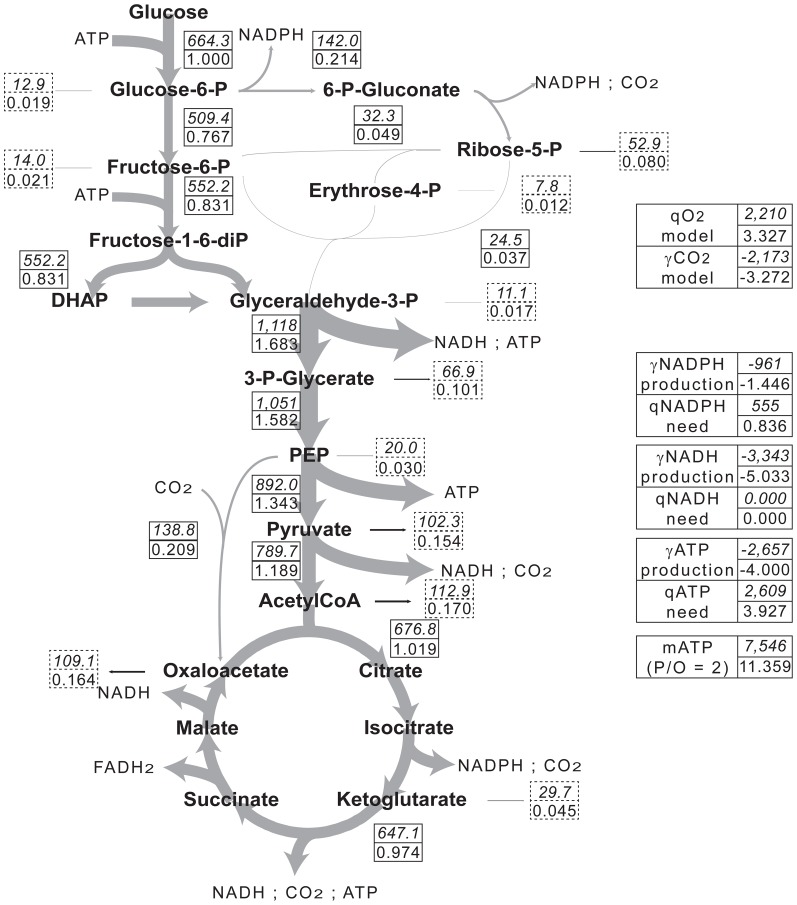
Carbon flux distribution in *S. coelicolor* M145. Grey arrows represent carbon fluxes within the central metabolism of *S. coelicolor* M145. Arrow widths are proportional to carbon fluxes. Flux values in the central metabolism are included in boxes with solid lines, flux values to biomass are included in boxes with dotted lines. The upper numbers represent average actual fluxes (μmol (g dry mass)^−1^ h^−1^), the lower number represent average normalized fluxes (μmol (μmol glucose)^−1^). Positive values correspond to consumption and negative values to production.

**Figure 2:**
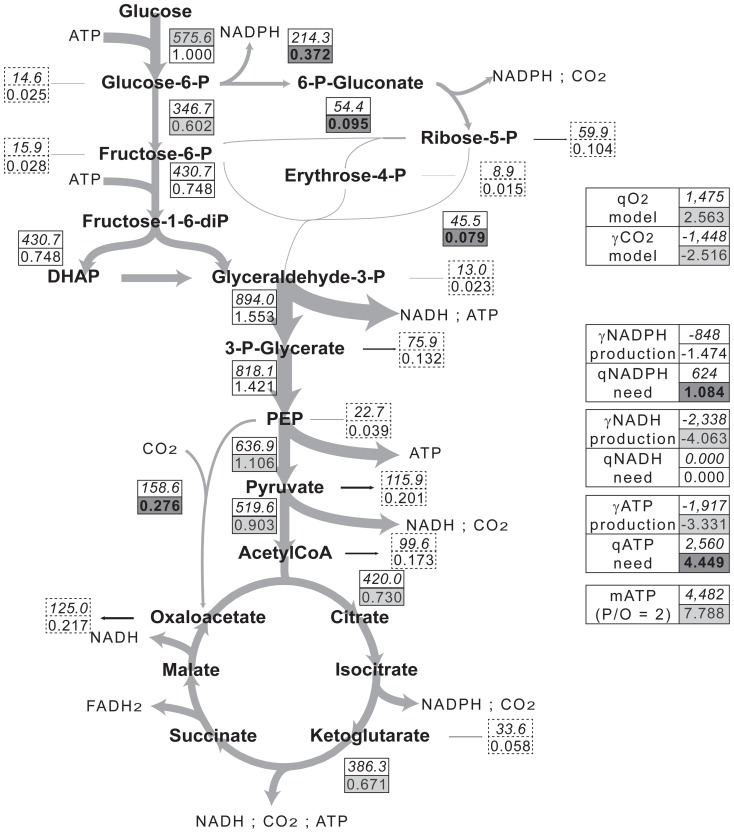
Carbon flux distribution in *S. coelicolor* M1146. Grey arrows represent carbon fluxes within the central metabolism of *S. coelicolor* M1146. Arrow widths are proportional to carbon fluxes. Flux values in the central metabolism are included in boxes with solid lines, flux values to biomass are included in boxes with dotted lines. The upper numbers represent average actual fluxes (μmol (g dry mass)^−1^ h^−1^), the lower number represent average normalized fluxes (μmol (μmol glucose)^−1^). Positive values correspond to consumption and negative values to production. Boxes with a dark grey shading indicate values significantly higher in M1146 than in M145. Boxes with a light grey shading indicate values significantly lower in M1146 than in M145.

Our results from Metabolic Flux Analysis and isotopic labeling were combined to draw a carbon flux map in M145 and M1146 during the exponential phase of growth (Fig. 1, Fig. 2). Fluxes were expressed as μmol (g cell dry weight)^−1^ h^−1^ or normalized to glucose uptake (μmol (μmol glucose)^−1^). Consistently, the committed step of PPP (glucose-6-phosphate dehydrogenase) was found to be associated with a flux of 0.372±0.002 µmol (μmol glucose)^−1^ in M1146, which is 1.7-fold higher than in M145 (0.214±0.003 µmol (μmol glucose)^−1^). In contrast, the commitment to the TCA was 1.019±0.02 µmol (μmol glucose)^−1^ in M145, that is, 1.4-fold higher than in M1146 (0.730±0.018 µmol (μmol glucose)^−1^). Precursor abstraction from primary carbon metabolism to biomass synthesis was larger in M1146 (total 1.019±0.018 µmol (μmol glucose)^−1^, giving 60±1% (molC mol^−1^C) of carbon uptake allocated to biosyntheses) compared to M145 (0.813±0.02 µmol (μmol glucose)^−1^, 47±1%). There was a much larger commitment of phospho*enol*pyruvate to carboxylation by the PEP carboxylase in M1146 and therefore, a larger anaplerotic oxaloacetate production to sustain the TCA.

Summing anabolic and catabolic fluxes for CO_2_ gave the total calculated CO_2_ evolution rate (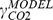) (Fig. 1, Fig. 2, right hand side). Our calculated fluxes may be compared to experimental values (Table 4, last line). There was good agreement between them, with a difference of 3.8±1% (M1146) and 2±1% (M145) only. Similarly, the total O_2_ consumption rate (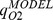) was computed from calculated fluxes and matched experimental *q_O2_* values very well (difference of less than 2.7±0.5%). Experimental and computed CO_2_ and O_2_ exchange rates gave respiratory quotients (*γ_CO2_*/*q_O2_*) of 0.982±0.002 for both strains, as would be expected during aerobic growth.

### Energy and redox balance

NADPH production was similar in both strains (Fig. 1, Fig. 2, right hand side). However, NADPH requirement for biomass biosynthesis was significantly higher for M1146 while in both, NADPH production exceeded needs. The production of NADH from catabolism and anabolism was lower (30±3%) in M1146 because the TCA was less active in that strain (see section Discussion 4). In M1146, the production of ATP from catabolism was lower (29±2%) than in M145 for the same reason. Contrary to M145, the production of ATP from catabolism was insufficient to cover ATP needs for biomass synthesis in M1146 (which are 13±0.1% higher than in M145, see section 4.3.). Nevertheless, a NADH-to-ATP conversion efficiency of 0.25 by the respiratory chain in M1146 would still cover ATP needs in that strain.

## Discussion

### Carbon fluxes in the central metabolism of *S. coelicolor*

The aim of this work was to reconstruct a comprehensive model of carbon flux distribution of primary carbon metabolism in *S. coelicolor* strains M145 and M1146, so as to better understand the consequences of the absence of antibiotic biosynthesis on central carbon metabolism. We used here both Metabolic Flux Analysis and ^13^C Metabolic Flux Analysis to solve fluxes across central carbon metabolism. Our analysis took advantage of published and experimental data on composition and reaction stoichiometries. As stated in section Results 4, this model seems valid since observed respiration rates (*q_O2_* and *γ_CO2_* values, Table 4) matched computed CO_2_ release and O_2_ consumption, with a respiratory quotient of nearly 1.

Nevertheless, Metabolic Flux Analysis is usually viewed as unlikely to give a completely faithful representation of the actual flux distribution, and consideration of areas in which thecarbon flux-balance model diverges from known metabolic behaviour may be potentially more informative. For example, the oxidative reactions of the oxidative pentose phosphate pathway typically carry no flux in flux-balance solutions of heterotrophic metabolism in plants [91], [92]. This discrepancy arises because mass-balance on NADPH can model the provision of NADPH by other possible pathways such as, for exemple, isocitrate dehydrogenase. Here, isotopic labeling with [^13^C-1] glucose was carried out to infer the metabolic commitment to the PPP, which is a source of NADPH. There was a very clear ^13^C-enrichment in TCA-derived amino acids (*m*_1_ and *m*_2_ much larger that they should be at natural ^13^C-abundance), allowing to quantify reliably the ^13^C-commitment to glycolysis and the TCA versus the PPP.

Using this approach, we determined a contribution of PPP to glucose catabolism in *S. coelicolor* M145 (22±0.3%, Fig. 1) close to that reported by Naiempoor and Mavituna (25.1%) for *S. coelicolor* during actinohodin production when the growth rate (*μ*) was near 0.04 h^−1^ [18].

In M1146, our estimate of the PPP contribution is 38±0.2% (Fig. 2), similar to that found at similar growth rates (*μ* near 0.04 h^−1^) in *S. lividans* (35%) during actinorhodin production [93], *Streptomyces noursei* (33%) during nystatin production [94] as well as *Streptomyces* C5 (30%) during ε-rhodomycinone production [39].

We postulate that the higher flux through the PPP in M1146 is the consequence of a higher NADPH demand to sustain both a higher growth rate (as previously suggested for *S. coelicolor* [93], [94]) and an increased triacylglycerol biosynthesis. Parenthetically, the higher NADPH demand might also contribute to coping with oxidative stress (see Discussion 4).

### Reductive power balance

It is believed that NADPH requirements and production should be balanced as cell metabolism reaches the steady state [95]. Here, in both strains, NADPH production exceeded NADPH needs (Fig. 1, Fig. 2, right hand side). If the NADPH content were steady, then the NADPH excess would have to be oxidized to NADP. In *S. coelicolor*, this might involve a nicotinamide nucleotide transhydrogenase, since our assays showed a thio-NAD reduction at the expense of NADPH. In their studies on actinorhodin production in an hyper-producing strain of *S. lividans*, Bruheim *et al*. [96] also suggested the involvement nicotinamide nucleotide transhydrogenase to balance excess NADPH production under conditions in which actinorhodin was synthesized.

Similarly, NADH production was larger than NADH needs and we assumed that this was compensated for by supplemental NADH oxidation through the respiratory chain. Other metabolic reactions that oxidise NADH might have also occurred, such as L-lactate dehydrogenase or alcohol dehydrogenase. However, such enzymatic activities are not likely since bacterial metabolism was aerobic under our conditions. In addition, we found no L-lactate and no ethanol at all in our experiments (data not shown).

### ATP needs, mATP and P/O

In strain M145, ATP generated by catabolism (4.000±0.043 µmol ATP (μmol glucose)^−1^) would cover ATP needs for anabolism (3.927±0.052 µmol ATP (μmol glucose)^−1^). To the contrary, an apparent ATP deficit (1.118±0.013 µmol ATP (μmol glucose)^−1^) would exist in strain M1146.

For M145 and M1146, 5.643±0.113 µmol NADH (μmol glucose)^−1^ and 4.453±0.089 µmol NADH (μmol glucose)^−1^ were generated in excess by metabolism and from putative NADPH transhydrogenation, respectively.

As the carbon balance do not close, we are aware that the calculation of ATP needs for anabolism may be affected if ATP is required for biosynthesis of the unidentified metabolites. This should not be the case for NADH, as NADH needs for anabolism are negligible (Table 3).

The amount of ATP generated by NADH reoxidation by the respiratory chain obviously depends upon the P/O ratio. Here, even a P/O ratio as low as 0.25 would have been sufficient to cover ATP needs in M1146.

If P/O ratios larger than 0.25 were taken into account, both the oxidation of total NADH to NAD by the respiratory chain and intrinsic metabolic phosphorylation (pyruvate kinase, *etc*.) would have produced an apparent ATP excess in both strains.

This apparent ATP excess, which is certainly balanced by ATP consumption for cell maintenance, may be denoted as mATP. mATP represents the energy demand for essential, non-growth-related processes, including maintenance of ionic potential across the cytoplasmic membrane, metabolite transport, cells homeostasis, macromolecules turn-over and others processes [82]. Since 80% of the actinorhodin pool in M145 were found in the extracellular compartment, a substantial part of mATP was probably dedicated to its excretion.

Calculated mATP strongly depends upon the P/O value used and in practice, the P/O value may vary substantially depending on growth conditions [97]. However, the P/O ratio chosen here has no consequence on the carbon flux distribution found in the present study, that is, any value of the P/O ratio above 0.25 would yield the same carbon flux pattern.

Here, taking into account a P/O value of 2, mATP values (on a biomass basis) would be 7.545±0.3 and 4.482±0.2 mmol ATP (g cell dry weight)^−1^ h^−1^ in M145 and M1146, respectively, that is, fell well within the range (4.52–9.64 mmol ATP (g cell dry weight)^−1^ h^−1^) of those found in various bacteria [98]–[102] and *S. coelicolor* [71] grown on glucose as a carbon source.

A standard way to estimate maintenance energy is to plot specific glucose uptake rate (mmol glucose (g cell dry weight)^−1^ h^−1^) as a function of the dilution rate *D* (h^−1^) in a chemostat [103], the *y* intercept being the maintenance energy value [104]. From consideration of published dilution rates [10], [19], [71], [93], [105], one may calculate *q_S_* values in *Streptomyces* and a value of 0.425±0.05 mmol glucose (g cell dry weight)^−1^ h^−1^ is found. Using our own experimental data, maintenance energy should correspond to 0.498±0.019 mmol glucose (g cell dry weight)^−1^ h^−1^ in M145 and 0.341±0.015 mmol glucose (g cell dry weight)^−1^ h^−1^ in M1146. Even though such values agree reasonably well with the average value from literature, an experimental determination of energy maintenance under our conditions remains to be determined.

### Alterations of metabolic fluxes in M1146 and their consequences

Since M145 produced solely actinorhodin from acetyl-CoA during its growth under our conditions, the genetic deletion of the cluster for actinorhodin biosynthesis in M1146 expectedly resulted in visible metabolic changes (Fig. 1, Fig. 2). Since acetyl-CoA is the main precursor of actinorhodin, acetyl-CoA accumulation could have been postulated in M1146. Nevertheless, acetyl-CoA accumulation is unlikely to occur for a number of biochemical [106], physiological [107], [108] and thermodynamical reasons.

Other evidences for the absence of acetyl-CoA accumulation came from the excretion of larger amounts of pyruvate in M1146 (167±11 µmol (g cell dry weight)^−1^ h^−1^) than in M145 (21±1 µmol (g cell dry weight)^−1^ h^−1^) during the stationary phase. In the absence of growth, acetyl-CoA could not be redirected to biomass in M1146 and accumulation was simply avoided by excreting its direct, freely diffusible precursor pyruvate.

During the exponential growth of M145, 80.4±1 µmol acetyl-CoA (g cell dry weight)^−1^ h^−1^ are dedicated to biomass production and 32.5±1 µmol acetyl-CoA (g cell dry weight)^−1^ h^−1^ to actinorhodin production. In M1146, with no other modification in glucose uptake and carbon flux distribution, a complete redirection of 32.5±1 µmol acetyl-CoA to biomass would result in a 40% increase of the specific growth rate (0.0552 h^−1^), a 40% increase of the anaplerotic flux and a decrease of *q_O2_* (1,581 µmol (g cell dry weight)^−1^ h^−1^) and *γ_CO2_* (1,534 µmol (g cell dry weight)^−1^ h^−1^) values in M1146.

However, the specific growth rate of M1146 was only 13±0.1% higher than that of M145 and the rate of glucose consumption was 13±0.5% lower. In M1146, there was therefore a slightly lower flux to acetyl-CoA synthesis due to the lower glucose consumption rate and as a result, the flux through the TCA and ATP production were lower (Fig. 2). Accordingly, *q_O2_* (1,475±0.05 µmol (g cell dry weight)^−1^ h^−1^) and *γ_CO2_* (1,448±0.05 µmol (g cell dry weight)^−1^ h^−1^) values were also lower.

The reasons of the limited increase in M1146 growth rate are not straightforward since *S. coelicolor* could grow with *μ* values up to 0.16 h^−1^, a high *q_S_* value (2 mmol (g cell dry weight)^−1^ h^−1^) and a low actinorhodin production rate (*γ_ACT_* = 6 nmol (g cell dry weight)^−1^ h^−1^) [19]. Three mechanisms at the origin of growth restriction are plausible:

First, in *Streptomyces peuceticus* var. *caesius* and *S. coelicolor*, a transcriptional regulation of glucose uptake rate has been demonstrated [36], [109], [110]. In M1146, a limitation of glucose entry mediated by an as yet unidentified regulatory protein (with acetyl-CoA or triacylglycerol as co-regulators) might have occurred.

Second, the absence of actinorhodin synthesis in M1146 might also cause growth limitation as a side effect. Indeed, quite recently, Shin *et al*. [111] demonstrated that the extracellular pool of actinorhodin was involved in the activation of the SoxR regulon in *S. coelicolor*. This regulon encodes proteins involved in the transport or the turn-over of small redox-active toxic compounds [112]. As described in *E. coli* by Fridovitch [113], such compounds might alter catalytic [4Fe-4S] centers of various dehydratases such as fumarase and aconitase in the TCA, or the dihydroxyacid dehydratase involved in the synthesis of abundant amino acids (Ile, Leu and Val), pantothenic acid and coenzyme A. Thus, under 70% of dissolved oxygen, the lack of actinorhodin production in M1146 might have lead to an increased intracellular oxidative stress, which in turn impeded the TCA, ATP synthesis, biomass synthesis and glucose consumption. The involvement of oxidative stress would be consistent with the increase in NADPH production by the PPP in M1146 (see Discussion 2).

Third, the carbon metabolism in M1146 partly compensated for the reduced growth rate (lower *μ*) and antibiotic synthesis by accumulating neutral lipidic compounds (triacylglycerol, see Results 1). In a similar molecular context, that is, in the *bldA* mutant of *S. coelicolor* unable to produce actinorhodin and undecylprodigiosin, Plaskitt and Chater [114] also demonstrated a higher triacylglycerol content. In fact, there seems to be a compromise between antibiotic and triacyglycerol production since *(i)* a higher actinorhodin production has been observed in the *sco0958* mutant impaired in triacylglycerol synthesis [115] and *(ii)* the simultaneous deletion of the gene clusters encoding 5-polyketide synthase and addition of a plasmid comprising the gene cluster encoding actinorhodin biosynthesis, resulted in a dramatic increase of actinorhodin production in *S. coelicolor* [116].

### M1146 as a super-host

M1146 was successfully used as a super-host for the production of actinorhodin, congocidin, chloramphenicol and caprazamycin after introduction of the corresponding gene clusters [10], [11]. However, the use of M1146 did not allow the efficient production of clorobiocin and coumermycin A1 [10].

The metabolic pathways associated with congocidin [117], chloramphenicol [118], caprazamycin [119], clorobiocin and coumermycin A1 production [120], [121] have all been elucidated. Precursor metabolites for the production of congocidin (3-phospho-glycerate, N-acetyl-glucosamine, sedoheptulose-7-phosphate and cytosine), chloramphenicol (shikimic acid, dichloro-acetyl-CoA), and caprazamycin (uridine, glycine, S-adenosyl-methionine, 3-methyl glutaryl-CoA, fatty acyl-CoA, per-methylated rhamnose) are all derived from glucose-1-phosphate, molecules from the pentose phosphate pathway, 3-phospho-glycerate and acetyl-CoA.

In the case of clorobiocin and coumermycin A1, precursors are, in that order of incorporation, prephenate (derived from both erythrose-4-phosphate and phospho*enol*pyruvate), noviose (derived from glucose-1-phosphate) and 3- or 5-methyl-pyrrole-2-carboxylic acids. These pyrrole groups originate from threonine (precursor oxaloacetate) or proline (precursor α-ketoglutarate). Flinspach *et al*. [10] have clearly shown that clorobiocin and coumermycin A1 production was unsuccessfull in M1146, the main products beeing aminocoumarin derivatives without any pyrrole group or not the expected ones.

Although we are aware of possible pleiotropic effects due to the introduction of foreign genes in a given genetic background, these results seem consistent with our carbon flux description in M1146, *i.e*., an enhanced flux through PPP, a moderately decreased glycolytic flux to acetyl-CoA, an increased acetyl-CoA extraction for triacylglycerol synthesis and a lower flux in the TCA. In clorobiocin and coumermycin A1 biosyntheses, the consumption of erythrose-4-phosphate and phospho*enol*pyruvate may exacerbate the flux impairement through the TCA, leading to the impossibility to produce a sufficient pyrrole content.

### Conclusion and perspectives

In this paper, we demonstrated that combining Metabolic Flux Analysis and ^13^C Metabolic Flux Analysis yielded an accurate description of carbon fluxes in *S. coelicolor* under steady-state conditions. Our work further suggested the key role of nicotinamide nucleotide transhydrogenase for redox homeostasis in *S. coelicolor.*

The main metabolic difference between M145 and M1146 consisted in an enhanced flux through the PPP in M1146, likely to face higher needs for NADPH in this strain. The metabolic differences between M145 and M1146 also highlighted the energetic cost for actinorhodin production and excretion in M145.

In other words, there would be a metabolic trade-off between instantaneous growth and the production of antibacterial compounds, which are likely to provide a competitive advantage [122] or to be involved in quorum sensing [112], [123], [124]. In other words, antibiotics are believed to help to defend the food source and to face bacterial competition in natural environments.

Our results also suggest a plausible competition between polyketide biosynthetic pathways for their common precursor, acetyl-CoA. Therefore, the deletion of polyketide antibiotic pathways might be instrumental in increasing acetyl-CoA availability and thus triacylglycerol, which is an important source of renewable oleochemicals. Conversely, the impairement of triacylglycerol synthesis might be of interest to improve polyketide antibiotics production yield.

In *Streptomyces*, antibiotics are usually produced in small amounts at the transition phase when bacterial growth slows down as a result of nutrient exhaustion, that is, not in steady-state conditions. New rapid sampling techniques [125], new modelling approaches and highly sensitive mass spectrometers make possible to carry out non-steady ^13^C Metabolic Flux Analysis [126], [127]. Together with our knowledge of *S. coelicolor* metabolism under steady-state conditions, it is now possible to study metabolic carbon fluxes during non-exponential growth stages. This will offer a better insight into the understanding of secondary metabolite biosyntheses under such conditions.

## Supporting Information

Table S1 Atom carbon transitions.
(DOC)

Table S2 Additional GC-MS analyses.
(DOC)

File S1 Supplementary Material and Methods.
(DOC)

File S2 Abbreviations and symbols.
(DOC)

File S3 The metabolic network of *S. coelicolor*.
(DOC)
