# Supplementary material for: Carbon-Flux Distribution within Streptomyces coelicolor Metabolism: A Comparison between the Actinorhodin-Producing Strain M145 and Its Non-Producing Derivative M1146
Source: PLoS One. 2013 Dec 23;8(12):e84151. doi: 10.1371/journal.pone.0084151 (PMC3871631; doi:10.1371/journal.pone.0084151)
Supplement: Table S1 — Atom carbon transitions. (DOC) [file pone.0084151.s001.doc]

**SUPPORTING TABLE S1**

**ATOM CARBON TRANSITIONS** PPP/Glycolysis flux determination.

|  | **rxnEq** | **rxnCTrans** |
| --- | --- | --- |
| R1 | GLC_EX + ATP = G6P | abcdef + X = abcdef |
| R2 | G6P = F6P | abcdef = abcdef |
| R3 | F6P + ATP = F16BP | abcdef + X = abcdef |
| R4 | F16BP = DHAP + GAP | abcdef = abc + def |
| R5 | DHAP = GAP | abc = cba |
| R6 | GAP = PGA + NADH + ATP | abc = abc + X + X |
| R7 | PGA = PEP | abc = abc |
| R8 | PEP = PYR + ATP | abc = abc + X |
| R9 | G6P = 6PG + NADPH | abcdef = abcdef + X |
| R10 | 6PG = R5P + CO2 + NADPH | abcdef = bcdef + a + X |
| R11 | R5P + R5P = E4P + F6P | abcde + fghij = bcde + fgahij |
| R12 | E4P + R5P = F6P + GAP | abcd + efghi = efabcd + ghi |
| R13 | PYR = AcCOA + CO2 + NADH | abc = bc + a + X |
| R14 | AcCOA + OAA = CIT | ab + cdef = fedbac |
| R15 | CIT = ISOCIT | abcdef = abcdef |
| R16 | ISOCIT = α KG + CO2 + NADPH | abcdef = abcde + f + X |
| R17 | α KG = 0.5 SUCC + 0.5 SUCC + CO2 + ATP + NADH | abcde = 0.5 bcde + 0.5 edcb + a + X + X |
| R18 | SUCC = MAL + FADH | abcd = abcd + X |
| R19 | MAL = OAA + NADH | abcd = abcd + X |
| R20 | PEP + CO2 = OAA + ATP | abc + d = abcd + X |
| R21 | G6P = G6P_B |  |
| R22 | F6P = F6P_B |  |
| R23 | GAP = GAP_B |  |
| R24 | PGA = PGA_B |  |
| R25 | PEP = PEP_B |  |
| R26 | PYR = PYR_B |  |
| R27 | R5P = R5P_B |  |
| R28 | E4P = E4P_B |  |
| R29 | AcCOA = AcCOA_B |  |
| R30 | α KG = α KG_B |  |
| R31 | OAA = OAA_B |  |
| R32 | CO2 = CO2_EX | a = a |
| R33 | α KG = GLU | abcde = abcde |
| R34 | α KG = PRO | abcde = abcde |
| R35 | OAA = THR | abcd = abcd |
| R36 | OAA = ASP | abcd = abcd |
| R37 | PYR = ALA | abc = abc |
| R38 | PYR + PYR = VAL + CO2 | abc + def = abefc + d |
| R39 | PGA = SER | abc = abc |
| R40 | SER = GLY + MeTHF | abc = ab + c |
| R41 | PYR + PYR = ISV + CO2 | abc + def = abefc + d |
| R42 | ISV + AcCOA = LEU + CO2 | abcde + fg = fgbcde + a |
| R43 | PYR + OAA = ILE + CO2 | abc + defg = debfgc + a |
| R44 | E4P + PEP = SHKM | abcd + efg = efgabcd |
| R45 | SHKM + PEP = CHRM | abcdefg + hij = abcdefghij |
| R46 | CHRM = PHE + CO2 | abcdefghij = hijbcdefg + a |

Carbon atom transitions are represented using letter code (rxnCTrans). For each metabolite, carbon atoms are identified using lower case letter to represent successive carbons atoms. RxnID is an ID for each reaction and rxnEq is the reaction. “_B” means drain to biomass and “_EX” means extracellular. More details about nomenclature are avalaible in the OpenFlux Manual.

**Non-conventional abbreviations**:

**6PG** : 6-Phosphogluconate ; **AcCoA** : AcetylCoA ; α **KG**: α-Ketoglutarate ; **CHRM** : Chorismic acid ; **CIT** : Citric acid ; **DHAP** : Dihydroxyacetone phosphate ; **E4P** : Erythrose-4-Phosphate ; **F16BP** : Fructose-1,6-bisphosphate ; **F6P** : Fructose-6-Phosphate ; **G6P** : Glucose-6-Phosphate ; **GAP** : Glyceraldehyde-3-Phosphate ; **GLC**: Glucose ; **ISOCIT** : Isocitric acid ; **ISV** : Isovaline ; **MAL** : Malic acid ; **MeTHF** : 5 ;10-methylenetetrahydrofolate or formylTHF ; **OAA** : Oxaloacetic acid ; **PEP** : Phosphoenolpyruvate ; **PGA** : 3-Phosphoglycerate ; **PYR** : Pyruvate ; **R5P** : Ribose-5-Phosphate ; **SHKM** : Shikimic acid ; **SUCC** : Succinic acid.
