# Supplementary material for: Carbon-Flux Distribution within Streptomyces coelicolor Metabolism: A Comparison between the Actinorhodin-Producing Strain M145 and Its Non-Producing Derivative M1146
Source: PLoS One. 2013 Dec 23;8(12):e84151. doi: 10.1371/journal.pone.0084151 (PMC3871631; doi:10.1371/journal.pone.0084151)
Supplement: Table S2 — Additional GC-MS analyses. (DOC) [file pone.0084151.s002.doc]

**SUPPORTING TABLE S2**

**ADDITIONAL GC-MS ANALYSES**

All the observed ^13^C % values below are determined with a standard error of about 0.35%.

| Compound | *m/z* intact^a^ | *m/z* decarboxylated^b^ | ^13^C intact^c^ | ^13^C decarboxylated^d^ | Pathway | Expected ^13^C intact^e^ | Expected ^13^C decarboxylated^f^ |
| --- | --- | --- | --- | --- | --- | --- | --- |
| Glutamate | 348 | 246 | 12.1 | 11.4 | GS^g^ | 12.4 | 15.3 |
| Glutamate | 348 | 246 | 12.1 | 11.4 | ED^h^ | 0 | 0 |
| Alanine | 218 | 116 | 8.8 | 9.6 | ED^h^ | 7.4 | 0 |
| Valine | 246 | 144 | 7.4 | 8.9 | ED^h^ | 4.9 | 0 |
| Pyroglutamate | 258 | 156 | 9.3 | 8.7 | GS^g^ | 12.4 | 15.3 |
| Pyroglutamate | 258 | 156 | 9.3 | 8.7 | ED^h^ | 0 | 0 |
| Proline | 244 | 142 | 7.9 | 8.9 | GS^g^ | 12.4 | 15.3 |
| Proline | 244 | 142 | 7.9 | 8.9 | ED^h^ | 0 | 0 |
| Histidine | 356 | 254 | 9.7 | 9.0 | C1/C6^i^ | 4.1 | 2.9 |

^a^ Monoisotopic *m/z* of intact ion. ^b^ Monoisotopic *m/z* of decarboxylated ion fragment. ^c^ Observed % 13C in the intact compound; the contribution of carbon atoms in the analyte from the chemical derivatization has been substracted: the value indicated here represents the % ^13^C of endogenous carbon atoms (metabolite). ^d^ Observed % 13C in the decarboxylated compound; the contribution of carbon atoms in the analyte from the chemical derivatization has been substracted: the value indicated here represents the % ^13^C of endogenous carbon atoms (metabolite). ^e^ Expected % ^13^C in the intact compound synthetised via the pathway considered. ^f^ Expected % ^13^C in the decarboxylated compound synthetised via the pathway considered. ^g^ Glyoxylic Shunt. ^h^ Entner-Doudoroff pathway. ^i^ C-1/C-6 isotopic equilibration in glucose.
