# Supplementary material for: Carbon-Flux Distribution within Streptomyces coelicolor Metabolism: A Comparison between the Actinorhodin-Producing Strain M145 and Its Non-Producing Derivative M1146
Source: PLoS One. 2013 Dec 23;8(12):e84151. doi: 10.1371/journal.pone.0084151 (PMC3871631; doi:10.1371/journal.pone.0084151)
Supplement: File S1 — Supplementary Material and Methods. (DOC) [file pone.0084151.s003.doc]

**SUPPORTING FILE S1**

**SUPPLEMENTARY MATERIAL AND METHODS**

**SFS1.1 Medium composition (bioreactor).**

TES (22.8 mmol L^-1^), Glucose (188.9 mmol L^-1^), Citric Acid.H_2_O (2.0 mmol L^-1^), NaCl (2.2 mmol L^-1^), KH_2_PO_4_ (11.0 mmol L^-1^), (NH_4_)_2_SO_4_ (43.0 mmol L^-1^), MgSO_4_.7H_2_O (1.7 mmol L^-1^), CaCl_2_.2H_2_O (0.2 mmol L^-1^), FeSO_4_.7H_2_O (137.6 μmol L^-1^), CuSO_4_.5H_2_O (12.0 μmol L^-1^), ZnSO_4_.7H_2_O (11.7 μmol L^-1^), MnSO_4_.H_2_O (33.9 μmol L^-1^), Na_2_MoO_4_.2H_2_O (1.6 μmol L^-1^), CoCl_2_.6H_2_O (3.2 μmol L^-1^), KI (1.5 μmol L^-1^), AlCl_3_.6H_2_O (1.3 μmol L^-1^), H_3_BO_3_ (2.5 μmol L^-1^), NiCl_2_.6H_2_O (1.6 μmol L^-1^), Thiamin/HCl (21.0 μmol L^-1^), Biotin (1.2 μmol L^-1^), Riboflavin (5.0 μmol L^-1^), Calcium Pantothenic acid (8.4 μmol L^-1^), Folic acid (0.6 μmol L^-1^), p-amino-benzoic acid (1.8 μmol L^-1^), Pyridoxine/HCl (9.7 μmol L^-1^), Nicotinamide (16.3 μmol L^-1^), Nicotinic acid (0.5 μmol L^-1^), 100.0 μL L^-1^ Antifoam 204 (Sigma Aldrich A8311) and 0.5 mL L^-1^ Pluronic F68 10 % (Sigma Aldrich P5556)

**SFS1.2 Nucleic acids extraction.**

10 mL of culture were centrifuged at 6,000 *g* for 10 min at 4 °C. The pellet was washed twice with 5 mL of cooled MgCl_2_ (1 mmol L^-1^), suspended in 2 mL of cooled MgCl_2_ (1 mmol L^-1^) and lyophilised.

20 to 60 mg of lyophilised sample were suspended in cooled HClO_4_ (0.25 mol L^-1^) and incubated for 20 min at 4 °C with gentle agitation in order to eliminate acid-soluble material (free sugars). The sample was centrifuged at 14,000 *g* for 5 min at 4 °C and the supernatant was discarded.

The nucleic acids were extracted with 4 mL HClO_4_ (0.5 mol L^-1^) and incubated for 15 min at 70 °C with gentle agitation. The supernatant was kept and nucleic acids were extracted two more times with 3 mL HClO_4_ (0.5 mol L^-1^) during 15 min at 70 °C. The three supernatants (10 mL) were pooled in order to quantify DNA and RNA [23].

**SFS1.3 DNA quantification.**

DNA was measured with the diphenylamine reagent, composed of 0.08 g acetaldehyde, 15 g diphenylamine, and 15 mL of concentrated H_2_SO_4_ in 1 L of cooled acetic acid. A sample (2 mL, diluted when necessary) was added to 2 mL of the diphenylamine reagent, incubated at 30 °C overnight and *A*_600_ was measured. A calibration curve was obtained with Salmon sperm DNA (Sigma Aldrich D1626) from samples containing 5 to 100 mg DNA mL^-1^ in the assay mixture [23].

**SFS1.4 RNA quantification.**

RNA was measured with the orcinol reagent, composed of 2 g orcinol, and 0.72 g FeCl_3_.6H_2_O in 1 L HCl (8 mol L^-1^). A sample (1 mL, diluted when necessary) was added to 3 mL of the orcinol reagent and incubated at 100 °C during 20 min. The sample was cooled on ice, 11 mL of n-butanol were added and *A*_672_ was measured. A calibration curve was obtained with RNA from samples containing 20 to 120 mg RNA mL^-1^ in the assay mixture [23].

**SFS1.5 Protein extraction and quantification.**

1 mL of culture was centrifuged at 6,000 *g* for 10 min at 4 °C. The pellet was washed twice with 1 mL of cooled Tris/HCl (50 mmol L^-1^) pH 8. After centrifugation, the pellet was frozen in liquid nitrogen.

The frozen pellet was suspended in 0.5 mL Tris/HCl (50 mmol L^-1^) pH 8, EDTA (1 mmol L^-1^), NaCl (10 mmol L^-1^), 2 mM MgCl_2_ (2 mmol L^-1^), DTT (1 mmol L^-1^), 5 % glycerol. The sample was sonicated 3 times with a Branson Sonifier 250 sonicator set at power 5, during 10 seconds at 30 second intervals.

0.1 mL of inhibitor mix (1 mL of protease inhibitor mix (Sigma Aldrich P8465), 0.1 mL Triton X-100 and 0.9 mL water) were added to the sample. 0.05 mL of 100-fold diluted benzonase (Sigma Aldrich E1014) were added. The sample was incubated for 15 min at RT with gentle agitation.

0.05 mL of 20 % SDS were added to the sample. The sample was incubated for 15 min at 4 °C with gentle agitation.

The extract was centrifuged at 14,000 *g* for 30 min at 4 °C. The protein concentrations were measured in the supernatant by the Bradford method using BSA as a standard.

**SFS1.6 Actinorhodin content.**

Actinorhodin content was measured according to Christiansen [25] as follows. 1 mL of culture was centrifuged at 13,000 *g* for 5 min at RT. The pellet was used to quantify intracellular actinorhodin and the supernatant to quantify extracellular actinorhodin.

To analyze the intracellular blue pigment (actinorhodin), the pellet was re-suspended in 1 mL KOH (1 mol L^-1^), thoroughly mixed for 20 min at 4 °C, and centrifuged 5 min at 3,000 *g*. The supernatant was collected and 500 μL HCl (3 mol L^-1^), were added to precipitate actinorhodin. This sample was incubated at 4 °C for 15 min, and then centrifuged 5 min at 13,000 *g.* The pellet was suspended in 1 mL KOH (1 mol L^-1^), and *A*_640_ was measured.

Actinorhodin concentrations were calculated according to the molar extinction coefficient (*ε*_640_ = 25,320 L mol^-1^ cm^-1^) of the pure compound in KOH (1 mol L^-1^).

To analyze the extracellular blue pigment (γ-actinorhodin), the supernatant was acidified with 500 μL HCl (3 mol L^-1^) to precipitate actinorhodin. This sample was incubated at 4 °C for 15 min, and then centrifuged 5 min at 13,000 *g.* The pellet was suspended in 1 mL KOH (1 mol L^-1^) and *A*_640_ was measured. Actinorhodin concentrations were calculated as before.

**SFS1.7 Undecylprodigiosin content.**

Undecylprodigiosin content was measured according to Tsao *et al*. [26] as follows. 1 mL of culture was centrifuged at 13,000 *g* for 5 min at RT and the pellet was used to quantify the intracellular red pigment undecylprodigiosin. The pellet was suspended in 1 mL of methanol, thoroughly mixed during 30 min at 4 °C, and centrifuged 5 min at 3,000 *g*. The supernatant was collected, and 1 mL HCl (1 mol L^-1^), was added. This sample was incubated at RT for 5 min, and then centrifuged 30 min at 13,000 *g.* 1 mL of supernatant was used to measure the *A*_530_.

Undecylprodigiosin concentrations were calculated taking into account the molar extinction coefficient (*ε*_530_ = 100,500 L mol^-1^ cm^-1^) for the pure compound in methanol/HCl (1 mol L^-1^) 1 : 1 v/v.

**SFS1.8 Calcium Dependent Antibiotic detection.**

Production of CDA was detected using a bioassay adapted from Lautru *et al*. [27]. The indicator bacterium *Micrococcus luteus* was spread on Oxoid nutrient agar plates containing 300 mmol L^-1^ FeCl_3_ and 15 mmol L^-1^ Ca(NO_3_)_2_. A 5-mm sterile filter paper disk was placed in the middle of each plate and impregnated with 10 μL of the supernatant to be tested. After overnight incubation at 37°C, growth inhibition was determined by measuring the diameter of the growth inhibition zone surrounding the disk.

**SFS1.9 Triacylglycerol content.**

The Triacylglycerol (TAG) content was quantified in lyophilized mycelia of *S. coelicolor* M145 and M114 using Fournier Transformed Infra Red spectroscopy (FTIR) using a Bruker Vertex 70 FTIR spectrometer with diamond ATR attachment (PIKE MIRacle crystal plate diamond/ZnSe) and MCT detector with a liquid nitrogen cooling system [28, 29]. Scanning was conducted from 4000 cm^-1^ to 400 cm^-1^ with a 4 cm^-1^ spectral resolution and with 100 repetitious scans averaged for each spectrum. Pure triacylglycerol was used as a standard.

The bands between 2959 cm^-1^ and 2852 cm^-1^ in FITR spectra correspond to the C-H stretching bands of the CH2 groups in fatty acid chains and the band near 1740 cm^-1^ correspond to the C=O stretching band of the carbonyl ester.

Since the protein content in biomass is known and since protein can be directly characterized by the amplitude of the Amide I absorption band (1650 cm^-1^), the TAG content of cells can be deduced from FTIR spectra.

**SFS1.10 Enzyme assays.**

40 mL of culture were harvested and bacterial cells were suspended in 10 mL Tris-Tricarballylic acid (15 mmol L^-1^) buffer pH 7.8, MgCl_2_ (10 mmol L^-1^), 10 % glycerol. RNase A (1 mg mL^-1^) and DNase I (1 mg mL^-1^) were added when needed. The sample was sonicated 8 times with a Branson Sonifier 250 sonicator set at power 2, during 20 seconds at 30 seconds intervals. Cell debris was removed by centrifuging at 10,000 *g* for 15 min at 4 °C. The resulting supernatant was used as the crude extract. The protein concentrations were measured by the Bradford method using BSA as a standard. All the enzyme activities were measured on a Beckmann DU7400 spectrophotometer at 28°C.

Glucose-6-phosphate dehydrogenase (EC 1.1.1.49) was assayed by a method based on that of Sugimoto and Shiio [30] in a reaction mixture containing Tris/HCl (100 mmol L^-1^) pH 7.8, MgCl_2_ (10 mmol L^-1^), with either NADP (0.5 mmol L^-1^) or NAD (0.5 mmol L^-1^) and glucose-6-phosphate (2 mmol L^-1^) as the substrate.

6-Phosphogluconate dehydrogenase (EC 1.1.1.44) was assayed by a method based on that of Sugimoto and Shiio [31] using the same reaction mixture as described above, except that 6-phosphogluconate (1 mmol L^-1^) was added as the substrate instead of glucose-6-phosphate.

Isocitrate dehydrogenase (EC 1.1.1.42) activity was assayed spectrophometrically as described by Nachlas *et al*. [32] in a reaction mixture containing Tris/HCl (100 mmol L^-1^) pH 7.8, MnCl_2_ (0.5 mmol L^-1^) with either NADP (0.5 mmol L^-1^) or NAD (0.5 mmol L^-1^) and isocitrate (2.5 mmol L^-1^) as the substrate.

Transhydrogenase (EC 1.6.1.2) was assayed in a mixture containing Tris/HCl (100 mmol L^-1^) pH 7.8, MgCl_2_ (0.5 mmol L^-1^) thio-NAD (0.1 mmol L^-1^) and NADPH (0.1 mmol L^-1^) as the substrate or in a mixture containing Tris/HCl (100 mmol L^-1^) pH 7.8, MgCl_2_ (0.5 mmol L^-1^) thio-NADP (0.1 mmol L^-1^) and NADH (0.1 mmol L^-1^) as the substrate [33].

The substrate-independent rates of cofactor reduction were followed and taken into account for the calculation of specific activities. The molar extinction coefficients used were *ε*_340_ = 6,220 L mol^-1^ cm^-1^ for NADH and NADPH, *ε*_398_ = 11,300 L mol^-1^ cm^-1^ for thio-NADH and thio-NADPH. All the activities were measured in triplicate. Mean values are given with confidence interval (*P* = 0.05).

**SFS1.11 Sample preparation and GC-MS analyses.**

**SFS1.11.1 Sample harvest.**

After 5 generations of exponential growth in the presence of 20 % [1-^13^C] glucose, cells from 50 mL of culture were harvested by centrifugation (5 min, 6,000 *g*, 4 °C). Cells were washed twice with 0.9 % NaCl, frozen in liquid nitrogen and then lyophilised overnight.

**SFS1.11.2 Cells hydrolysis.**

10 mg of lyophilised cells were re-suspended in 2 mL of HCl (6 mol L^-1^) and incubated at 105 °C over night to hydrolyse proteins. HCl was then evaporated at 95 °C to sample dryness.

**SFS1.11.3 Metabolites extraction.**

20 mg of the lyophilised pellet were re-suspended in 1 mL of extraction solution (80 % methanol, 20 % water). 1 mL of internal standard 1 (a-amino-n-butyric acid at 0.2 mmol L^-1^in extraction solution) was added. The sample was vortexed for 20 s three times with 5 mm diameter silver beads of. The sample was centrifuged (15 min, 13,000 *g*, 4 °C). 90 μL of internal standard 2 (adonitol at 2 mmol L^-1^ in extraction solution) were added to 1.8 mL of supernatant. The sample was centrifuged again (15 min, 13,000 *g*, 4 °C). Three aliquots of 0.5 mL were evaporated overnight to complete dryness.

**SFS1.11.4 Amino acids derivatization (MSTFA).**

100 μL of methoxyamine solution (20 mg mL^-1^ methoxyamine in pyridine) were added to the dried hydrolysate. After vortexing for 10 sec, 50 μL of supernatant were incubated at 30 °C during 90 min under constant agitation. Then, 80 μL of N-methyl-N-(trimethylsilyl) trifluoroacetamide (MSTFA) were added to the sample. The mixture was vortexed 30 sec and incubated at 37 °C for 30 min under constant agitation. The sample was finally incubated at RT for 120 min without agitation. 10 μL of an alcane preparation in hexane (7 mmol L^-1^ of Decane, 1.4 mmol L^-1^ of Pentadecane, Octadecane, Nonadecane, Docosane, Octacosane, Dotriacontane and Hexatriacontane) were added as internal standards for retention time calibration.

**SFS1.11.5 GC-MS analyses.**

1 μL of derivatized amino acid sample was injected into an Agilent 6890N gas chromatograph (GC) with a RTX-5 column linked to integra-Guard (30 m x 0.25 mm i.d x 10 m integrated guard column) using helium (0.7 mL min^-1^) as a gas vector. The temperature remained at 80°C for 2 min. A ramp of temperature was then begun, starting at 80°C and ending at 330°C with an increment of 15°C min^-1^. The temperature remained at 330 °C for 6 min. The GC was coupled with a Pegasus III MS time-of-flight mass spectrometer (solvant delay 315 s; acquisition frequency 20 Hz; detector voltage 1600 V; ionisation source –70 eV at 200°C).

Peak integration was performed using LECO Pegasus software. As automated peak integrations have been found to be occasionally unreliable, hand-made controls and/or corrections were systematically performed for each analysis.
