# Supplementary material for: Carbon-Flux Distribution within Streptomyces coelicolor Metabolism: A Comparison between the Actinorhodin-Producing Strain M145 and Its Non-Producing Derivative M1146
Source: PLoS One. 2013 Dec 23;8(12):e84151. doi: 10.1371/journal.pone.0084151 (PMC3871631; doi:10.1371/journal.pone.0084151)
Supplement: File S2 — Abbreviations and symbols. (DOC) [file pone.0084151.s004.doc]

**SUPPORTING FILE S2**

**ABBREVIATIONS AND SYMBOLS**

$f_{g}^{\text{in}}$: Air flow in the bioreactor (L min^-1^)

$F_{i}^{X}$: Flux from metabolite *i* for biomass synthesis (μmol (g cell dry weight)^-1^ h^-1^)

$F_{i}^{\text{ACT}}$: Flux from metabolite *i* for ACT synthesis (μmol (g cell dry weight)^-1^ h^-1^)

$F_{i}^{\text{RED}}$: Flux from metabolite *i* for RED synthesis (μmol (g cell dry weight)^-1^ h^-1^)

$F_{i}^{\text{CDA}}$: Flux from metabolite *i* for CDA synthesis (μmol (g cell dry weight)^-1^ h^-1^)

$F_{i}^{\text{SEC}}$: Flux from metabolite *i* for organic acid secretion (μmol (g cell dry weight)^-1^ h^-1^)

*l_i_*: Need of precursor *i* for ACT synthesis (μmol)

*m_i_*: Need of precursor *i* for RED synthesis (μmol)

*n_i_*: Need of precursor *i* for CDA synthesis (μmol)

*m*_0_: Peak intensity of the monoisotopic ion-fragment (*e.g*. for glutamate-TMS, *m/z* = 348)

*m*_1_: Peak intensity of the *m*+1 ion-fragment (*e.g.* for glutamate-TMS, *m/z* = 349)

*m*_2_: Peak intensity of the *m*+2 ion-fragment (*e.g.* for glutamate-TMS, *m/z* = 350)

*MW_X_*: Molecular weight (g mol^-1^)

*p_i_*: Need of precursor *i* for biomass synthesis (μmol (g cell dry weight)^-1^)

*q_S_*: Substrate consumption rate (μmol (g cell dry weight)^-1^ h^-1^)

*γ_ACT_*: ACT production rate (μmol (g cell dry weight)^-1^ h^-1^)

*γ_RED_*: RED production rate (μmol (g cell dry weight)^-1^ h^-1^)

*γ_KET_*: α-ketoglutarate production rate (μmol (g cell dry weight)^-1^ h^-1^)

*γ_PYR_*: Pyruvate production rate (μmol (g cell dry weight)^-1^ h^-1^)

*γ_CO2_*: Experimental CO_2_ evolution (μmol (g cell dry weight)^-1^ h^-1^)

*q_O2_*: Experimental O_2_ consumption rate (μmol (g cell dry weight)^-1^ h^-1^)

: Predicted CO_2_ production rate (μmol (g cell dry weight)^-1^ h^-1^)

$q_{O2}^{\text{MODEL}}$: Predicted O_2_ consumption rate (μmol (g cell dry weight)^-1^ h^-1^)

$q_{i}^{\text{MODEL}}$: Predicted consumption rate for metabolite *i* (μmol (g cell dry weight)^-1^ h^-1^)

*QR*: Respiratory quotient (*q*_CO2_ (*q*_O2_) ^-1^)

*T°C*: Temperature (° Celsius)

*TMS*: Trimethylsilyl derivative

: Total anabolic flux for metabolite *i* (μmol (g cell dry weight)^-1^ h^-1^)

$v_{i}^{\text{CATA}}$: Total catabolic flux for metabolite *i* (μmol (g cell dry weight)^-1^ h^-1^)

*Y_X/S_*: Yield of substrate conversion to biomass (mol-C biomass (mol-C substrate)^-1^)

*Y_ACT/S_*: Yield of substrate conversion to ACT (mol-C ACT (mol-C substrate)^-1^)

*Y_RED/S_*: Yield of substrate conversion to RED (mol-C RED (mol-C substrate)^-1^)

*Y_CDA/S_*: Yield of substrate conversion to CDA (mol-C CDA (mol-C substrate)^-1^)

*Y_KET/S_*: Yield of substrate conversion to α-ketoglutarate (mol-C KET (mol-Csubstrate)^-1^)

*Y_PYR/_*: Yield of substrate conversion to pyruvate (mol-C PYR (mol-C substrate)^-1^)

*Y_CO2/S_*: Yield of substrate conversion to CO_2_ (mol-C CO_2_ (mol-C substrate)^-1^)

*Y_O2/S_*: Yield of substrate conversion to O_2_ (mol O_2_ (mol-C substrate)^-1^)

*μ* : Growth rate constant (h^-1^)

: CO_2_ concentration in exhaust gas phase (%)

${\%O2}_{g}^{\text{out}}$: O_2_ concentration in exhaust gas phase (%)
