# Supplementary material for: Carbon-Flux Distribution within Streptomyces coelicolor Metabolism: A Comparison between the Actinorhodin-Producing Strain M145 and Its Non-Producing Derivative M1146
Source: PLoS One. 2013 Dec 23;8(12):e84151. doi: 10.1371/journal.pone.0084151 (PMC3871631; doi:10.1371/journal.pone.0084151)
Supplement: File S3 — The metabolic network of S. coelicolor. (DOC) [file pone.0084151.s005.doc]

**SUPPORTING FILE S3**

**THE METABOLIC NETWORK OF *S. COELICOLOR***

**SFS3.1 AMINO ACIDS**

**Alanine**

Pyr + NADPH + NH_3_ ⇔ (Alanine) + NADP^+^ + H_2_O

**Arginine**

AcCoA + α KG + OAA + 5 ATP + 4 NADPH + 4 NH_3_ + CO_2_ ⇔ (Arginine) + CoASH + 4 ADP + AMP + 4 Pi + PPi + 4 NADP^+^ + acetic acid + fumaric acid + H_2_O

**Asparagine**

OAA + 2 ATP + NADPH + 2 NH_3_ ⇔( Asparagine) + ADP + AMP + PPi + Pi + NADP^+^ + H_2_O

**Aspartic acid**

OAA + NADPH + NH_3_ ⇔ (Aspartate) + NADP^+^ + H_2_O

**Cysteine**

PGA + AcCoA + NADPH + NAD^+^ + NH_3_+ SH_2_ ⇔ (Cysteine) + Pi + NADH + NADP^+^ + acetic acid + CoASH

**Glutamic acid**

α KG + NADPH + NH_3_ ⇔ (Glutamic acid) + NADP^+^ + H_2_O

**Glutamine**

α KG + ATP + NADPH + 2 NH_3_ ⇔ (Glutamine) + ADP + Pi + NADP^+^ + H_2_O

**Glycine**

PGA + NAD^+^ + NADPH + NH_3_ + THF ⇔ (Glycine) + Pi + NADH + NADP^+^ + MeTHF + H_2_O

**Histidine**

R5P + 4 ATP + 2 NAD^+^ + NADPH + 2 NH_3_ ⇔ (Histidine) + 2 ADP + AMP + 3 Pi + 2 PPi + 2 NADH + NADP^+^ + AICAR

**Isoleucine**

OAA + Pyr + 2 ATP + 5 NADPH + NH_3_ ⇔ (Isoleucine) + 2 ADP + 2 Pi + 5 NADP^+^ + 2 H_2_O + CO_2_

**Leucine**

2 Pyr + AcCoA + NAD^+^ + 2 NADPH + NH_3_ ⇔ (Leucine) + NADH + 2 NADP^+^ + 2 CO_2_ + CoASH + H_2_O

**Lysine**

Pyr + OAA + ATP + 4 NADPH + 2 NH_3_ + Succ CoA ⇔ (Lysine) +ADP + Pi + 4 NADP^+^ + CO_2_ + CoASH + succinic acid + 2 H_2_O

**Methionine**

PGA + AcCoA + OAA + ATP + NAD^+^ + 4 NADPH + Succ CoA + NH_3_ + SH_2_ + MeTHF ⇔ (Methionine) + Pyr + ADP + 2 Pi + NADH + 4 NADP^+^ + THF + 2 CoASH + succinic acid + acetic acid

**Phenylalanine**

E4P + 2 PEP+ ATP + 2 NADPH + NH_3_ ⇔ (Phenylalanine) + ADP + 4 Pi + 2 NADP^+^ + CO_2_ + 2 H_2_O

**Proline**

α KG + ATP + 3 NADPH + NH_3_ ⇔ (Proline) + ADP + Pi + 3 NADP^+^ + 2 H_2_O

**Serine**

PGA + NAD^+^ + NADPH + NH_3_ ⇔ (Serine) + Pi + NADH + NADP^+^

**Threonine**

OAA + 2 ATP + 3 NADPH + NH_3_ ⇔ (Threonine) + 2 ADP + 2 Pi + 3 NADP^+^

**Tryptophan**

R5P + E4P + PGA + 2 PEP + 3 ATP + NAD^+^ + 2 NADPH + 2 NH_3_ ⇔ (Tryptophan) + Pyr + GAP + 2 ADP + AMP + 6 Pi + PPi + NADH + 2 NADP^+^+ CO_2_ + 3 H_2_O

**Tyrosine**

E4P + 2 PEP + ATP + NAD^+^ + 2 NADPH + NH_3_ ⇔ (Tyrosine) + ADP + 4 Pi + NADH + 2 NADP^+^ + CO_2_ + H_2_O

**Valine**

2 Pyr + 2 NADPH + NH_3_ ⇔ (Valine) + 2 NADP^+^ + CO_2_ + 2 H_2_O

**SFS3.2 RIBONUCLEOTIDES TRIPHOSPHATES**

**ATP**

R5P + PGA + 2 OAA + 10 ATP + NAD^+^ + 3 NADPH + 5 NH_3_ + CO_2_ + MeTHF + GTP ⇔ (ATP) + 9 ADP + AMP + 9 Pi + PPi + NADH + 3 NADP^+^ + THF + 2 fumaric acid + GDP + 4 H_2_O

**GTP**

R5P + PGA + OAA + 12 ATP + 2 NAD^+^ + 2 NADPH + 5 NH_3_ + CO2 + MeTHF + 2 H_2_O ⇔ (GTP) + 10 ADP + 2 AMP + 9 Pi + 2 PPi + 2 NADH + 2 NADP^+^ + THF + fumaric acid

**CTP**

R5P + OAA + 8 ATP + NAD^+^ + NADPH + 3 NH_3_ ⇔ (CTP) + 7 ADP + AMP + 5 Pi + PPi + NADH + NADP^+^ + 3 H_2_O

**UTP**

R5P + OAA + 6 ATP + NAD^+^ + NADPH + 2 NH_3_ ⇔ (UTP) + 5 ADP + AMP + 3 Pi + PPi + NADH + NADP^+^ + 2 H_2_O

**SFS3.3 DEOXYRIBONUCLEOTIDES TRIPHOSPHATES**

**dATP**

R5P + PGA + 2 OAA + 10 ATP + NAD^+^ + 3 NADPH + 5 NH_3_ + CO_2_ + MeTHF + GTP + E-(SH)_2_ ⇔ (dATP) + 9 ADP + AMP + 9 Pi + PPi + NADH + 3 NADP^+^ + THF + 2 fumaric acid + GDP + E-S_2_ + 4 H_2_O

**dGTP**

R5P + PGA + OAA + 12 ATP + 2 NAD^+^ + 2 NADPH + 5 NH_3_ + CO2 + MeTHF + 2 H_2_O + E-(SH)_2_ ⇔ (dGTP) + 10 ADP + 2 AMP + 9 Pi + 2 PPi + 2 NADH + 2 NADP^+^ + THF + fumaric acid + E-S_2_

**dCTP**

R5P + OAA + 8 ATP + NAD^+^ + NADPH + 3 NH_3_ + E-(SH)_2_ ⇔ (dCTP) + 7 ADP + AMP + 5 Pi + PPi + NADH + NADP^+^ + E-S_2_ + 2 H_2_O

**dTTP**

R5P + OAA + 10 ATP + NAD^+^ + NADPH + 2 NH_3_ + E-(SH)_2_ ⇔ (dTTP) + 9 ADP + AMP + 5 Pi + 2 PPi + NADH + NADP^+^ + E-S_2_

**SFS3.4 PHOSPHOLIPIDS**

**Phosphatidyl ethanolamine**

GAP + PGA + NAD^+^ + 2 NADPH + NH_3_ + CTP+ 2 R-ACP ⇔ (PE) + PPi + Pi + NADH + 2 NADP^+^ + CO_2_+ CMP + 2 ACP + 4 H_2_O

**Phosphatidyl glycerol**

2 GAP + 2 NADPH + CTP + 2 R-ACP ⇔ (PG) + Pi + PPi + 2 NADP^+^ + CMP + 2 ACP + 3 H_2_O

**Cardiolipin**

3 GAP + 3 NADPH + 2 CTP + 4 R-ACP + 2 H_2_O ⇔ (cardiolipin) + 2 PPi + 3 NADP^+^ + 2 CMP + 4 ACP + Pi

**C14**

7 AcCoA + 6 ATP + 12 NADPH ⇔ (C14) + 6 ADP + 6 Pi + 12 NADP^+^ + 7 CoASH + 5 H_2_O

**C15**

Pyr + 5 AcCoA + OAA + 7 ATP + NAD^+^ + 14 NADPH ⇔ (C15 anteiso) + 7 ADP + 7 Pi + NADH + 14 NADP^+^+ 2 CO_2_ + 5 CoASH + 3 H_2_O

**C16**

8 AcCoA + 7 ATP + 14 NADPH ⇔ (C16) + 7 ADP + 7 Pi + 14 NADP^+^ + 8 CoASH + 6 H_2_O

**C17**

Pyr + 6 AcCoA + OAA + 8 ATP + NAD^+^ + 16 NADPH ⇔ (C17 anteiso) + 8 ADP + 8 Pi + NADH + 16 NADP^+^ + 2 CO_2_ + 6 CoASH + 7 H_2_O

**C18 : 1**

9 AcCoA + 8 ATP + 2 NADH + 16 NADPH + O_2_ ⇔ (C18: 1) + 8 ADP + 8 Pi + 2 NAD^+^+ 16 NADP^+^ + 9 CoASH + 9 H_2_O

**SFS3.5 TRIACYLGLYCEROL**

**Triacylglycerol**

GAP + NADPH + 3 R-SCoA + H_2_O ⇔ (triacylglycerol) + Pi + NADP^+^ + 3 CoASH

**Glycerol-3-phosphate**

GAP + NADPH ⇔ (Glycerol-3-P) + NADP^+^

**C14**

7 AcCoA + 6 ATP + 12 NADPH ⇔ (C14)-SCoA + 6 ADP + 6 Pi + 12 NADP^+^ + 6 CoASH + 5 H_2_O

**C15**

Pyr + 5 AcCoA + OAA + 7 ATP + NAD^+^ + 14 NADPH ⇔ (C15 anteiso)-SCoA + 7 ADP + 7 Pi + NADH + 14 NADP^+^+ 2 CO_2_ + 4 CoASH + 3 H_2_O

**C16**

8 AcCoA + 7 ATP + 14 NADPH ⇔ (C16)-SCoA + 7 ADP + 7 Pi + 14 NADP^+^ + 7 CoASH + 6 H_2_O

**C17**

Pyr + 6 AcCoA + OAA + 8 ATP + NAD^+^ + 16 NADPH ⇔ (C17 anteiso)-SCoA + 8 ADP + 8 Pi + NADH + 16 NADP^+^ + 2 CO_2_ + 5 CoASH + 7 H_2_O

**C18 :1**

9 AcCoA + 8 ATP + 2 NADH + 16 NADPH + O_2_ ⇔ (C18:1)-SCoA + 8 ADP + 8 Pi + 2 NAD^+^+ 16 NADP^+^ + 8 CoASH + 9 H_2_O

**SFS3.6 SOLUBLE MOLECULES**

**NAD**

R5P + GAP + OAA + 4 ATP + NADPH + 2 NH_3_ + O_2_ ⇔ (NAD^+^) + ADP + 2 AMP + 2 Pi + 3 PPi + NADP^+^ + CO_2_ + 2 H_2_O

**NADP**

R5P + GAP + OAA + 5 ATP + NADPH + 2 NH_3_ + O_2_ ⇔ (NADP^+^) + 2 ADP + 2 AMP + 2 Pi + 3 PPi + NADP^+^ + CO_2_ + 2 H_2_O

**CoA**

PGA + 2 Pyr + AcCoA + OAA + 4 ATP + NAD^+^ + 4 NADPH + 2 NH_3_ + MeTHF + H_2_S + CTP ⇔ (CoA) + 2 ADP + AMP + Pi + 3 PPi + NADH + 4 NADP^+^ + 3 CO_2_ + THF + CoASH + CMP + acetic acid + H_2_O

**Menaquinone 8**

E4P + 8 GAP + 2 PEP + 7 Pyr + α KG + 10 ATP + 9 NADPH + 8 CTP + SAM ⇔ (MK8) + 9 ADP + AMP + 4 Pi + 17 PPi + 9 NADP^+^+ 17 CO_2_ + 8 CMP + SAH + H_2_O

**THF**

R5P + E4P + PGA + 2 PEP + α KG + OAA + 16 ATP + 2 NAD^+^ + 5 NADPH + 7 NH_3_ + CO_2_ + MeTHF ⇔ (THF) + Pyr + 13 ADP + 3 AMP + 16 Pi + 4 PPi + 2 NADH + 5 NADP^+^ + THF + fumaric acid + Formate + glycoaldehyde + H_2_O

**FMN**

3 R5P + PGA + OAA + 13 ATP+ 2 NAD^+^ + 3 NADPH + 4 NH_3_ + MeTHF ⇔ (FMN) + 11 ADP + 2 AMP + 12 Pi + 3 PPi + 2 NADH + 3 NADP^+^ + 2 CO_2_ + THF + fumaric acid + H_2_O

**FAD**

3 R5P + PGA + OAA + 14 ATP+ 2 NAD^+^ + 3 NADPH + 4 NH_3_ + MeTHF ⇔ (FMN) + 11 ADP + 2 AMP + 12 Pi + 4 PPi + 2 NADH + 3 NADP^+^ + 2 CO_2_ + THF + fumaric acid + H_2_O

**ATP**

R5P + PGA + 2 OAA + 10 ATP + NAD^+^ + 3 NADPH + 5 NH_3_ + CO_2_ + MeTHF + GTP ⇔ (ATP) + 9 ADP + AMP + 9 Pi + PPi + NADH + 3 NADP^+^ + THF + 2 fumaric acid + GDP + 4 H_2_O

**ADP**

R5P + PGA + 2 OAA + 9 ATP + NAD^+^ + 3 NADPH + 5 NH_3_ + CO_2_ + MeTHF + GTP ⇔ (ATP) + 8 ADP + AMP + 9 Pi + PPi + NADH + 3 NADP^+^ + THF + 2 fumaric acid + GDP + 4 H_2_O

**SFS3.7 PEPTIDOGLYCAN**

**UDP-N-Acetylmuramic acid**

R5P + OAA + F6P + PEP + AcCoA + 7 ATP + NAD^+^ + 2 NADPH + 3 NH_3_ ⇔ (UDP-NAM) + 6 ADP + AMP + 5 Pi + 2 PPi + NADH + 2 NADP^+^ + CoASH + H_2_O

**UDP-N-Acetylglucosamine**

R5P + F6P + AcCoA + OAA + 7 ATP + NAD^+^ + NADPH + 3 NH_3_ ⇔ (UDP-NAG) + 6 ADP + AMP + PPi + 4 Pi + NADH + NADP^+^ + CoASH + 2 H_2_O

**Alanine**

Pyr + NADPH + NH_3_ ⇔ (Alanine) + NADP^+^ + H_2_O

**Diaminopimelic acid**

Pyr + OAA + ATP + 4 NADPH + 2 NH_3_ + Succ CoA ⇔ (DAP) +ADP + Pi + 4 NADP^+^ + CoASH + succinic acid + 2 H_2_O

**D-Glutamic acid**

α KG + NADPH + NH_3_ ⇔ (Glutamic acid) + NADP^+^ + H_2_O

**Glycine**

PGA + NAD^+^ + NADPH + NH_3_ + THF ⇔ (Glycine) + Pi + NADH + NADP^+^ + MeTHF + H_2_O

**Bactoprenol Phosphate**

11 Pyr + 11 GAP + 11 ATP + 11 NADPH + 11 CTP ⇔ (bactoprenolP) + 11 ADP + 21 PPi + Pi + 11 NADP^+^ + 11 CO_2_ + 11 CMP

**SFS3.8 CELLWALL CARBOHYDRATES**

**UDP-N-Acetylglucosamine**

R5P + F6P + AcCoA + OAA + 7 ATP + NAD^+^ + NADPH + 3 NH_3_ ⇔ (UDP-NAG) + 6 ADP + AMP + PPi + 4 Pi + NADH + NADP^+^ + CoASH + 2 H_2_O

**UDP-Galactose**

R5P + G6P + OAA + 6 ATP + NAD^+^ + NADPH + 2 NH_3_ ⇔ (UDP-Galactose) + 5 ADP + AMP + 2 PPi + 3 Pi + NADH + NADP^+^ + 2 H_2_O

**SFS3.9 TEICHOIC ACIDS**

**Teichoic acid**

10 G6P + 2 F6P+ 12 GAP + 2 AcCoA + 2 ATP + 12 NADPH +2 NH_3_ + 12 CTP + 12 UTP ⇔ (Teichoic acid) + 2 ADP + 2 Pi + 24 PPi + 12 NADP^+^ + 12 CMP + 11 UDP + UMP + 23 H_2_O

**Lysine**

Pyr + OAA + ATP + 4 NADPH + 2 NH_3_ + Succ CoA ⇔ (Lysine) +ADP + Pi + 4 NADP^+^ + CO_2_ + CoASH + succinic acid + 2 H_2_O

**SFS3.10 ANTIBIOTICS**

**Actinorhodin (ACT)**

16 AcCoA + 15 ATP + 4 NAD^+^ + 12 NADPH + 5.5 O_2_ + 4 FMNH2 ⇔ (ACT) + 15 ADP + 15 Pi + 4 NADH + 12 NADP^+^+ 16 CoASH + 4 FMN^+^ + 13 H_2_O

**Undecylprodigiosin (RED)**

2 PGA + 8 AcCoA + α KG + 11 ATP + 2 NAD^+^ + 15.5 NADPH + 3 NH_3_ + THF + 2 FAD^+^ + 0.5 FMN^+^+ SAM ⇔ (RED) + 11 ADP + 13 Pi + 2 NADH + 15.5 NADP^+^ + 2 CO_2_ + MeTHF + 8 CoASH + 2 FADH2 + 0.5 FMNH2 + SAH + 13 H_2_O

**Calcium Dependent Antibiotic form 1b (CDA1b)**

2 R5P + 3 E4P+ 4 PGA + 6 PEP + 4 AcCoA+ 5 OAA + 2 α KG + 26 ATP + 5 NAD^+^ + 20 NADPH + 14 NH_3_ + 4 O_2_ + THF + FADH2 + FMN^+^ + (PLP) + HSACP ⇔ (CDA1b) + 2 GAP + 2 Pyr + 11 ADP + 15 AMP + 47 Pi + 3 PPi + 5 NADH + 20 NADP^+^ + 5 CO2 + MeTHF + 4 CoASH + succ + FAD^+^ + FMNH2 + (PMP) + H_2_O_2_ + AcSACP + 27 H_2_O

**Calcium Dependent Antibiotic form 3b (CDA3b)**

2 R5P + 3 E4P + 4 PGA + 6 PEP + 4 AcCoA + 5 OAA + 2 α KG + 25 ATP + 5 NAD^+^ + 20 NADPH + 14 NH_3_ + 4 O_2_ + THF + FADH2 + FMN^+^+ (PLP) + HSACP ⇔ (CDA3b) + 2 GAP + 2 Pyr + 10 ADP + 15 AMP + 47 Pi + 3 PPi + 5 NADH + 20 NADP^+^ + 5 CO_2_ + MeTHF + 4 CoASH + succ + FAD^+^ + FMNH2 + (PMP) + H_2_O_2_ + AcSACP + 27 H_2_O

**Calcium Dependent Antibiotic form 3a (CDA3a)**

2 R5P + 3 E4P + 4 PGA + 6 PEP + 4 AcCoA + 5 OAA + 2 α KG + 25 ATP + 5 NAD^+^ + 20 NADPH + 14 NH_3_ + 5 O_2_ + THF + FADH2 + FMN^+^+ (PLP) + HSACP ⇔ (CDA3b) + 2 GAP + 2 Pyr + 10 ADP + 15 AMP + 47 Pi +3 PPi + 5 NADH + 20 NADP^+^ + 5 CO_2_ + MeTHF + 4 CoASH + succ + FAD^+^ + FMNH2 + (PMP) + 2 H_2_O_2_ + AcSACP + 27 H_2_O

**Calcium Dependent Antibiotic form 2b (CDA2b)**

2 R5P + 3 E4P + 4 PGA + 6 PEP + 4 AcCoA + 5 OAA + 2 α KG + 26 ATP + 5 NAD^+^ + 20 NADPH + 14 NH_3_ + 4 O_2_ + THF + FADH2 + FMN^+^+ (PLP) + SAM + HSACP ⇔ (CDA2b) + 2 GAP + 2 Pyr + 11 ADP + 15 AMP + 47 Pi + 3 PPi + 5 NADH + 20 NADP^+^ + 5 CO_2_ + MeTHF + 4 CoASH + succ + FAD^+^ + FMNH2 + (PMP) + H_2_O_2_ + SAH + AcSACP + 27 H_2_O

**Calcium Dependent Antibiotic form 2a (CDA2a)**

2 R5P + 3 E4P + 4 PGA + 6 PEP + 4 AcCoA + 5 OAA + 2 α KG + 26 ATP + 5 NAD^+^ + 20 NADPH + 14 NH_3_ + 5 O_2_ + THF + FADH2 + FMN^+^+ (PLP) + SAM + HSACP ⇔ (CDA2a) + 2 GAP + 2 Pyr + 11 ADP + 15 AMP + 47 Pi + 3 PPi + 5 NADH + 20 NADP^+^ + 5 CO_2_ + MeTHF + 4 CoASH + succ + FAD^+^ + FMNH2 + (PMP) + 2 H_2_O_2_ + SAH + AcSACP + 27 H_2_O

**Calcium Dependent Antibiotic form 4b (CDA4b)**

2 R5P + 3 E4P + 4 PGA + 6 PEP + 4 AcCoA + 5 OAA + 2 α KG + 25 ATP + 5 NAD^+^ + 20 NADPH + 14 NH_3_ + 4 O_2_ + THF + FADH2 + FMN^+^+ (PLP) + SAM + HSACP ⇔ (CDA4b) + 2 GAP + 2 Pyr + 10 ADP + 15 AMP + 47 Pi +3 PPi + 5 NADH + 20 NADP^+^ + 5 CO_2_ + MeTHF + 4 CoASH + succ + FAD^+^ + FMNH2 + (PMP) + H_2_O_2_ + SAH + AcSACP + 27 H_2_O

**Calcium Dependent Antibiotic form 4a (CDA4a)**

2 R5P + 3 E4P + 4 PGA + 6 PEP + 4 AcCoA + 5 OAA + 2 α KG + 25 ATP + 5 NAD^+^ + 20 NADPH + 14 NH_3_ + 5 O_2_ + THF + FADH2 + FMN^+^+ (PLP) + SAM + HSACP ⇔ (CDA4a) + 2 GAP + 2 Pyr + 10 ADP + 15 AMP + 47 Pi + 3 PPi + 5 NADH + 20 NADP^+^ + 5 CO_2_ + MeTHF + 4 CoASH + succ + FAD^+^ + FMNH2 + (PMP) + 2 H_2_O_2_ + SAH + AcSACP + 27 H_2_O

**Non-conventional abbreviations**:

**6PG** : 6-Phosphogluconate ; **AcCoA** : AcetylCoA ; **ACP** : Acylcarrierprotein ; **AcSACP** : Acetyled acylcarrierprotein ; **AICAR** : Aminoimidazole carboxamide ribonucleotide ; α **KG**: α-Ketoglutarate ; **C14** : Fatty acid 14 carbons  ; **C15** : Fatty acid 15 carbons  ; **C16** : Fatty acid 16 carbons  ; **C17** : Fatty acid 17 carbons  ; **C18 : 1** : Fatty acid 18 carbons with insaturated bond ; **CHRM** : Chorismic acid ; **CIT** : Citric acid ; **CoASH** : Reduced coenzymeA ; **DAP** : Diaminopimelic acid ; **DHAP** : Dihydroxyacetonephosphate ; **E4P** : Erythrose-4-Phosphate ; **E-(SH)_2_**: thioredoxin**; F16BP** : Fructose-1,6-bisphosphate ; **F6P** : Fructose-6-Phosphate ; **G6P** : Glucose-6-Phosphate ; **GAP** : Glyceraldehyde-3-Phosphate ; **HSACP** : Acylcarrierprotein ; **ISOCIT** : Isocitric acid ; **ISV** : Isovaline ; **MAL** : Malic acid ; **MeTHF** : 5 ;10-methylenetetrahydrofolate or formylTHF ; **MK8** : Menaquinone 8 ; **OAA** : Oxaloacetic acid ; **PE** : Phosphatidyl ethanolamine ; **PEP** : Phospho*enol*pyruvate ; **PGA** : 3-Phosphoglycerate ; **PLP** : Pyridoxal ; **PMP** : Pyridoxamine ; **PYR** : Pyruvate ; **R5P** : Ribose-5-Phosphate ; **SAH** : S-adenosylhomocysteine ; **SAM** : S-adenosylmethionine ; **SHKM** : Shikimic acid ; **SUCC** : Succinic acid ; **Succ CoA** : SuccinylCoA ; **THF** : Tetrahydrofolate ; **TTP** : Thymidine triphosphate ; **UDP-NAG** : UDP-N-Acetylglucosamine ; **UDP-NAM** : UDP-N-Acetylmuramic acid
